# Unraveling the Genetic Elements Involved in Shoot and Root Growth Regulation by Jasmonate in Rice Using a Genome-Wide Association Study

**DOI:** 10.1186/s12284-019-0327-5

**Published:** 2019-09-04

**Authors:** Huong Thi Mai To, Hieu Trang Nguyen, Nguyet Thi Minh Dang, Ngan Huyen Nguyen, Thai Xuan Bui, Jérémy Lavarenne, Nhung Thi Phuong Phung, Pascal Gantet, Michel Lebrun, Stephane Bellafiore, Antony Champion

**Affiliations:** 1 University of Science and Technology of Hanoi (USTH), Vietnam Academy of Science and Technology (VAST), LMI-RICE2, 18 Hoang Quoc Viet, Cau Giay district, Hanoi, Vietnam; 2 Institut de Recherche pour le Développement (IRD), Université de Montpellier, UMR DIADE, UMR IPME, UMR LSTM, Montpellier, France; 3 Agricultural Genetics Institute, LMI-RICE2, Hanoi, Vietnam

**Keywords:** Jasmonate, Plant development, Growth inhibition, Genome-wide association studies, *Oryza sativa*

## Abstract

**Background:**

Due to their sessile life style, plant survival is dependent on the ability to build up fast and highly adapted responses to environmental stresses by modulating defense response and organ growth. The phytohormone jasmonate plays an essential role in regulating these plant responses to stress.

**Results:**

To assess variation of plant growth responses and identify genetic determinants associated to JA treatment, we conducted a genome-wide association study (GWAS) using an original panel of Vietnamese rice accessions. The phenotyping results showed a high natural genetic variability of the 155 tested rice accessions in response to JA for shoot and root growth. The level of growth inhibition by JA is different according to the rice varieties tested. We conducted genome-wide association study and identified 28 significant associations for root length (RTL), shoot length (SHL), root weight (RTW), shoot weight (SHW) and total weight (TTW) in response to JA treatment. Three common QTLs were found for RTL, RTW and SHL. Among a list of 560 candidate genes found to co-locate with the QTLs, a transcriptome analysis from public database for the JA response allows us to identify 232 regulated genes including several JA-responsive transcription factors known to play a role in stress response.

**Conclusion:**

Our genome-wide association study shows that common and specific genetic elements are associated with inhibition of shoot and root growth under JA treatment suggesting the involvement of a complex JA-dependent genetic control of rice growth inhibition at the whole plant level. Besides, numerous candidate genes associated to stress and JA response are co-located with the association loci, providing useful information for future studies on genetics and breeding to optimize the growth-defense trade-off in rice.

**Electronic supplementary material:**

The online version of this article (10.1186/s12284-019-0327-5) contains supplementary material, which is available to authorized users.

## Background

Rice is the main staple food for over half of the world’s population. The demand on the quality and quantity of rice is now more and more strongly emphasized due to increasing global population as well as in the context of climate change.

In order to withstand both biotic and abiotic stresses, plants evolved efficient and sophisticated systems including an inducible defense system mediated by jasmonate (JA) (Nahar et al. 2012; Okada et al. 2015; Khan et al. 2016). This phyto-hormone synthesized from the fatty acid linolenic acid, plays important roles in plants, for example, in regulating development, growth and defense response to biotic stresses. The function of JA relies on the core, conserved signaling module, which constitutes the amino acid-conjugated bioactive compound JA-Ile, the JA receptor CORONATINE INSENSITIVE 1 (COI1) protein, the co-receptor and repressor JASMONATE-ZIM domain protein (JAZ), and the transcription factors (such as MYC2, MYC3, MYC4, MYC5…) (Chini et al. 2007; Katsir et al. 2008; Ye et al. 2009; Kazan and Manners 2013; Yan et al. 2016; Howe et al. 2018). In response to the developmental cues or stress signals, JA-Ile is accumulated and percieved by the COI1 receptor, the JAZ repressors is recruited for ubiquitination and degradation through the 26S proteasome manner, thereby relieving the repression of transcription factors and enabling the expression of JA-responsive genes and JA responses (Wasternack and Hause 2013). Besides these main components, the JAZ repressor also interact with other proteins, such as TOPLESS and NINJA (NOVEL INTERACTER of JAZ), to repress the transcription factor MYC2 (Pauwels et al. 2010; Gasperini et al. 2015). The rice genome consists of three *OsCOI* genes (designated as *OsCOI1a*, *OsCOI1b*, and *OsCOI2*), in which only *OsCOI1a* and *OsCOI1b* could rescue the sterility phenotype in Arabidopsis *coi1* mutant (Lee et al. 2013). The number of *OsJAZ* and *OsNINJA* gene are also higher than that in Arabidopsis with 15 *OsJAZ* genes (designated as *OsJAZ1* to *OsJAZ15*) (Ye et al. 2009), and four *OsNINJA* (Kashihara et al. 2019), as compared to 13 *JAZ* and a single *NINJA* in Arabidopsis, respectively. Among these genes, the function of some of them have been investigated (Ye et al. 2009; Yamada et al. 2012; Toda et al. 2013; Cai et al. 2014; Hori et al. 2014; Wu et al. 2015; Hakata et al. 2017; Li et al. 2017; Kashihara et al. 2019). The functional homologous of MYC2, OsMYC2, was also characterized as the master regulator that involved in numerous response in rice (Uji et al. 2016; Ogawa et al. 2017a; Ogawa et al. 2017b; Uji et al. 2017). Although it is generally postulated that JA impacts the energy balance of plants between defense and development during stress condition, the mechanisms underlying this regulation are still not fully understood (Wasternack 2007; Nahar et al. 2011). When the JA-dependent defense system is continuously activated, growth of the plant can be severely affected (Vos et al. 2013; Huot et al. 2014). As an example, repeated wounding of *Arabidopsis* cotyledons inhibits cell division and cell elongation causing a reduction in root length in a JA-dependent manner (Gasperini et al. 2015). In *Arabidopsis* and rice, interaction between JA and gibberellin signaling cascade regulate the growth-defense dynamics by which plant prioritize JA-dependent defense response over growth (Yang et al. 2012). Recently, Marcelo Campos et al demonstrated that growth-defence antagonism in *Arabidopsis* is regulated through a JA-dependent transcriptional network that restricts growth upon defense activation by JA (Campos et al. 2016). These contrasting activities of the hormone imply a broader role for the JA in regulating a compromise between growth and defense, thereby optimizing plant fitness in rapidly changing environments. Therefore, it is interesting to identify specific genes that could optimize the defense system of plants with less impact on growth.

Since JA is a central hormone in various stress signaling processes in plants, applying JA is a simplified way to mimic different types of stress. For example, the exogenous application of JA has been widely used to screen mutants collection affected in stress and development responses of plants in general and in rice in particular (Ye et al. 2009; Chan et al. 2010; Nahar et al. 2012). In *Arabidopsis*, the JA receptor COI1 protein has been identify following the phenotyping of mutagenized seedlings treated by coronatine, a structural homologue of the bioactive jasmonoyl-isoleucine hormone (Feys et al. 1994). In addition to COI1, other major JA-signaling proteins such as a JAZ transcriptional repressor and the transcription factor AtMYC2 have been identified based on JA treated mutant population (Lorenzo et al. 2004; Chini et al. 2007). Beside, exogenous JA application has also been widely used to study complex treats such as dynamic and architectures of JA regulatory network and hormones interaction involved in root architecture or leaf growth inhibition (Sun et al. 2009; Noir et al. 2013; Hickman et al. 2017).

In order to explore the diversity of rice growth inhibition in response to JA, 155 accessions of a rice collection were treated with exogenous JA. This collection represent a core panel of Vietnamese rice landraces that originate from different geographical locations and are adapted to different agrosystems including irrigated, rain-fed, lowland, upland or mangrove ecosystems. In accordance with their genetic nature, they can be divided into 3 types: *Indica* (89% - mostly lowland rice), *Japonica* (9.5% - mostly upland rice) and other (1.5%) (Bui 2010). They possess many precious traits including resistance to biotic stress, such as blast, blight, and brown plant hopper, and abiotic stress, such as tolerance to salinity or drought as well as submergence conditions (Bùi and Nguyễn 2003; Bui 2010). Some accessions that have good tolerance to various types of stress have been identified and used as sources of stress tolerance in breeding to create new varieties. However, even though the Vietnamese rice resources likely carry many valuable alleles that can be beneficial for agronomy, they remain insufficiently explored and utilized (Courtois et al. 1997; Bui 2010).

This collection has also already been genotyped by GBS with 21,613 SNP (Phung et al. 2014). Phung et al have successfully used this collection to identify new QTLs associated with root development in non-stress conditions using a genome-wide association study (GWAS) (Phung et al. 2016). Furthermore, GWAS has been conducted using this core panel of rice landraces to reveals new association loci controlling panicle morphological traits (Ta et al. 2018) and tolerance to water deficit during vegetative stage demonstrating the valuable genetic resource of this rice collection (Hoang et al. 2019).

While responses to jasmonate have been studied considerably, it remains unclear how these responses are modulated by the genetic diversity. Here, we assess variation of root and shoot traits upon treatment with JA in the 155 rice accessions. We conducted genome-wide association studies and identified 28 significant associations with rice growth inhibition by JA, including three common QTLs associated to root and shoot growth inhibition traits. Using functional annotation and expression analyses we retrieved 560 genes linked to the most significant markers, and found 42% of the candidate genes to be responsive to jasmonate, indicating that among these genes are possible new players in jasmonate growth inhibition response pathway.

## Results

### Dose-Effect of JA on Rice Plantlet Growth

To study the dose-response to JA, in vitro experiments were carried out with *Oryza sativa indica* accession IR64 in order to define the optimal JA concentration for treatments. Eight concentrations of JA as 0 μM (control), 0.5 μM, 1 μM, 2.5 μM, 5 μM, 7.5 μM, 10.5 μM and 12.5 μM were chosen after several trials (data not shown). After 7 days of exposure to JA, root length (RTL), shoot length (SHL), root weight (RTW), shoot weight (SHW) and total weight (TTW) were evaluated as parameters to analyze the dose-effect of JA on rice growth.

**Fig. 1:**
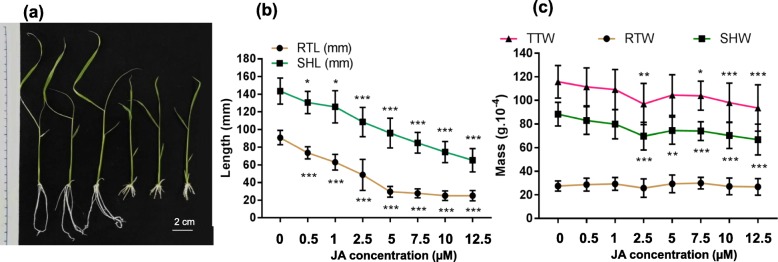
Effect of the JA concentration on rice plantlets (IR64) development. Five parameters were collected from 7 day-old plantlets after JA treatment and growth in 1/2 MS with or without JA. Phenotype of the plantlet IR64 (**a**), plants on 0 μM medium were on the left, plants on 5 μM medium were on the right; Data represent means of three biological replicates (±SD) of root and SHL (**b**); TTW- RTW and SHW (**c**) from 15 to 17 plantlets of each condition. Student’s t- test was used to assess the differences between the plant grown under untreated condition (0 μM) versus under different concentration of JA. The significant is shown with *p*-values: (*) *p* < 0.05, (**) *p* < 0.01, (***) *p* < 0.001

As shown in Fig. 1a and b, treatment with 0.5 μM of JA was sufficient to reduce RTL and SHL compared to the untreated condition (*P* < 0.05). This effect was highly significant (*P* < 0.0001) from the JA concentration of 2.5 μM for these both parameters. At 2.5 μM JA, a distinct difference was also observed for SHW (*P* < 0.0001) and TTW (*P* < 0.0001), as shown in Fig. 1c. However, we observed a remarkable reduction for SHL and RTL at 5 μM JA of approximately 40% and 60%, respectively. Therefore, to ensure that we could observe the diversity of response within varieties, the phenotypes of these accessions after treatment with 5 μM JA for a total 10 days from the germination stage, including 7 days of JA exposure, were recorded. A first trial of experiment with 10 Vietnamese rice accessions that represent the genetic diversity of the Vietnamese rice accessions were chosen based on the study of Phung et al (Phung et al. 2014) was conducted. The Vietnamese rice panel was divided into 2 main sub-panels: *Indica* and *Japonica*. The *indica* subpanel was further divided into six populations (I1 to I6) and the *japonica* subpanel was divided into 4 populations (J1 to J4). This screening with 6 *Indica* accessions and 4 *Japonica* accessions was performed to evaluate the diversity of our rice accessions response to JA exogenous treatment. The phenotyping dataset indicated an obvious difference between treated and non-treated plants for all 10 varieties, and these representative accessions respond significantly differently to JA (5 μM) (Additional file 1: Figures S1-S3 and Table S1). This preliminary study showed that 5 μM of JA was suitable to highlight varied growth response with different genotypes. In addition, IR64 was used as an internal control in the full panel phenotyping,

### Trait Heritability and Genetic Variability of 155 Rice Accessions

A list of the 155 accessions and their relevant information used in this study is presented in Additional file 2: Table S2. An analysis on the phenotypic variation and broad sense heritability is presented in Table 1. To confirm whether the variation exhibited in a certain trait could be attributed to genetic variation, the heritability coefficient was calculated according to the formula given by (Wray and Visscher 2008). From the result obtained, in both control and treatment conditions with the broad-sense heritability of all traits ranging from 0.90 to 0.96, the variations of the 5 studied traits were caused mostly by genetic polymorphisms, with well-controlled environmental factors. Hence, the quality of the phenotype dataset was sufficient for further study with genome-wide association mapping.

**Table 1:** phenotypic variation and broad sense heritability for each trait

Trait	Mean None treated	CV None treated	*H*^*2*^ None treated	Mean JA treated	CV JA treated	*H*^*2*^ JA treated
RTL (mm)	62.73986	7.857902	0.95	20.23247	7.858259	0.9
SHL (mm)	161.9466	28.58407	0.96	94.68829	24.91233	0.95
RTW (mg)	23.42427	5.550502	0.9	24.10098	6.765589	0.92
SHW (mg)	83.87246	20.16218	0.95	61.34949	16.19226	0.95
TTW (mg)	107.2967	25.03204	0.95	85.45047	21.83225	0.95

### Phenotypic Diversity of the Vietnamese Rice Collection in Response to 5 μM JA Treatment

**Fig. 2:**
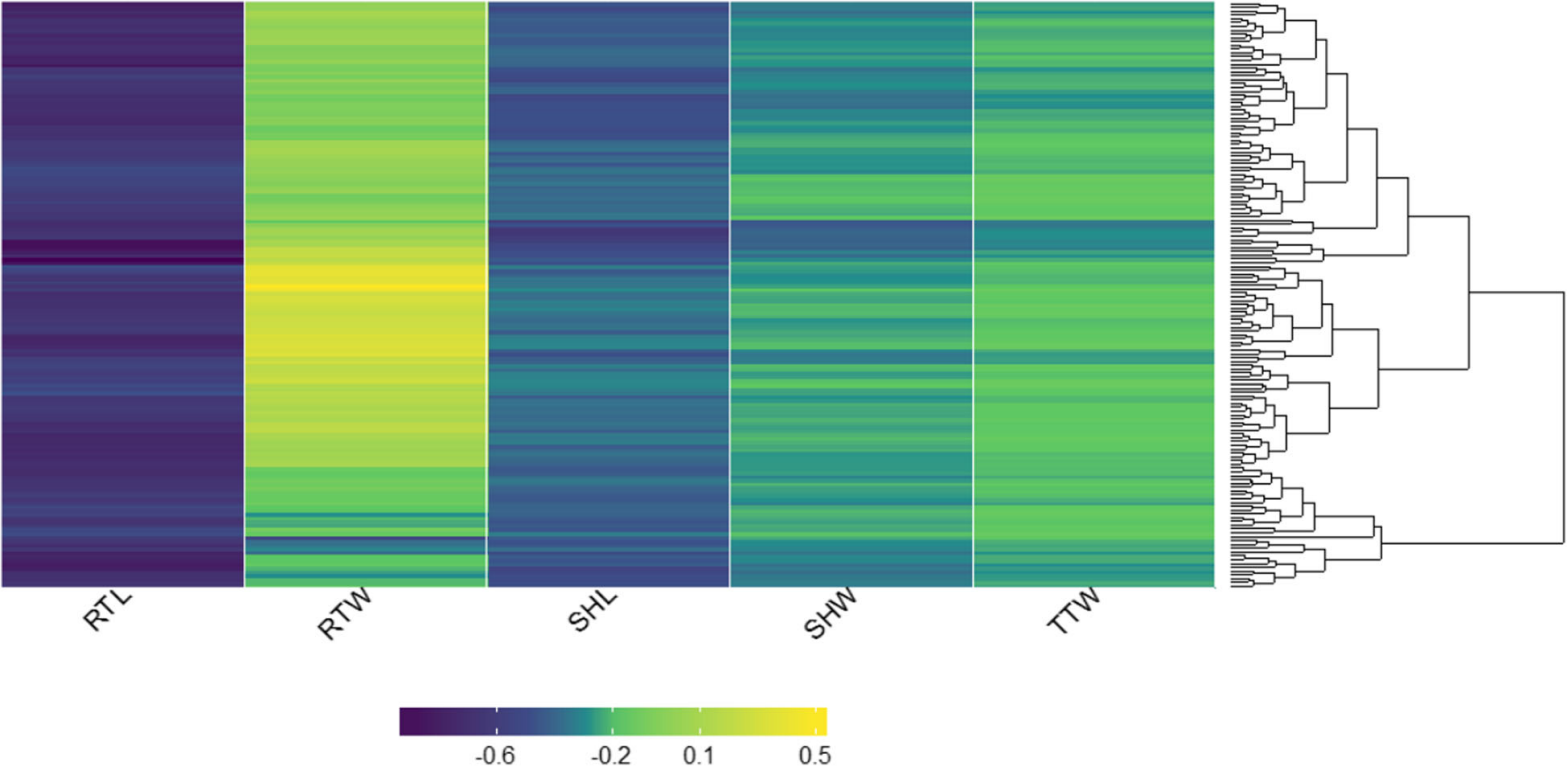
Comparative visualization of genotype-phenotype relationships of 155 rice accessions. Ratio of variation or Delta value reflect for impact of JA on 5 traits including root length (RTL), root weight (RTW), shoot length (SHL), shoot weight (SHW) and total weight (TTW) respectively. The heat map was created using the “3dheatmap” package in R. The negative value represent the inhibition effect of JA to the plant growth, and the positive value represent the induction effect of JA

Effects of 5 μM JA on the phenotypic variation of the growth traits for 155 rice accessions were evaluated and illustrated in Fig. 2 and Additional file 3: Table S3.

The 155 rice accessions show considerable diversity in response to JA for all traits, predicting the diversity in response to environmental stresses. Except for RTW, exogenous JA treatment has a negative impact on all analyzed traits causing the limited growth of rice accessions. JA treatment has a significant negative effect on RTL and shoots length for all accessions. There are some rice accessions that are particularly affected by JA, such as accession G40 in which the RTL was inhibited up to 97% compared to the control condition, while the least susceptible variety, such as accession G52, showed RTL inhibition of 48% in response to JA. JA also inhibits shoot growth with a slightly lower ratio than that of the root, the most susceptible G132 with 66% SHL reduction and the least susceptible G146 with only 30% reduction, compared to plantlets in control condition. In contrast to the length parameters, the RTW and TTW of the plant are also affected but do not follow the same inhibition trend. Particularly, over half of accessions tend to increase the RTW when plants are stressed with exogenous JA. For example, with accession G65, although the root length is reduced by 68%, the dry weight of roots increased by nearly 50% when this variety was treated while for accession G223 both of RWT and RTL reduced by 55% and 62%, respectively. This can be explained by the increase in the number of crown roots as well as the root’s diameter in these accessions (data not shown). Taken together, these results indicated a high phenotypic diversity among the rice core panel in response to JA.

### Phenotypic Heterogeneity Among *Indica* and *Japonica* Sub-Groups and Among Ecosystem

**Fig. 3:**
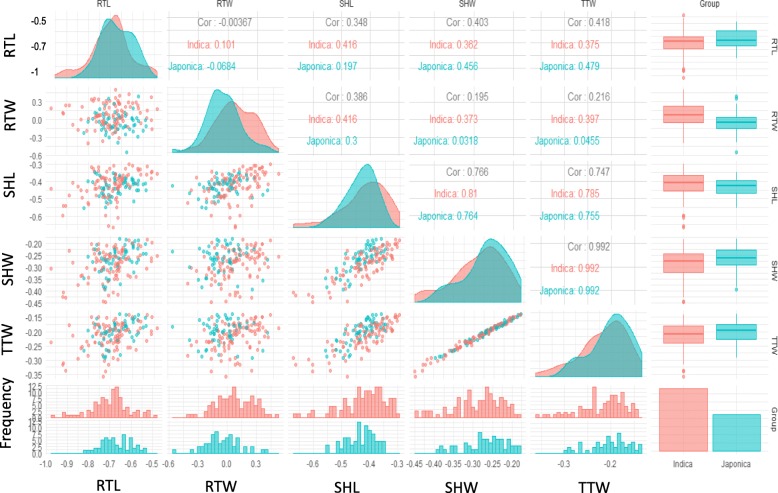
Phenotypic heterogeneity among *Indica* and *Japonica* sub-groups and pair correlation of 5 traits based on the effect of JA. *Indica* group indicate in pink and Japonica group colored as blue cyan. The distribution of value of each trait in each panel was represented by histogram at the bottom of figure and their median values were shown by boxplot in the right hand. The values pair correlation of traits as well as their plots was shown symmetrically in the figure. The figure was created using “ggpairs” package in R

**Fig. 4:**
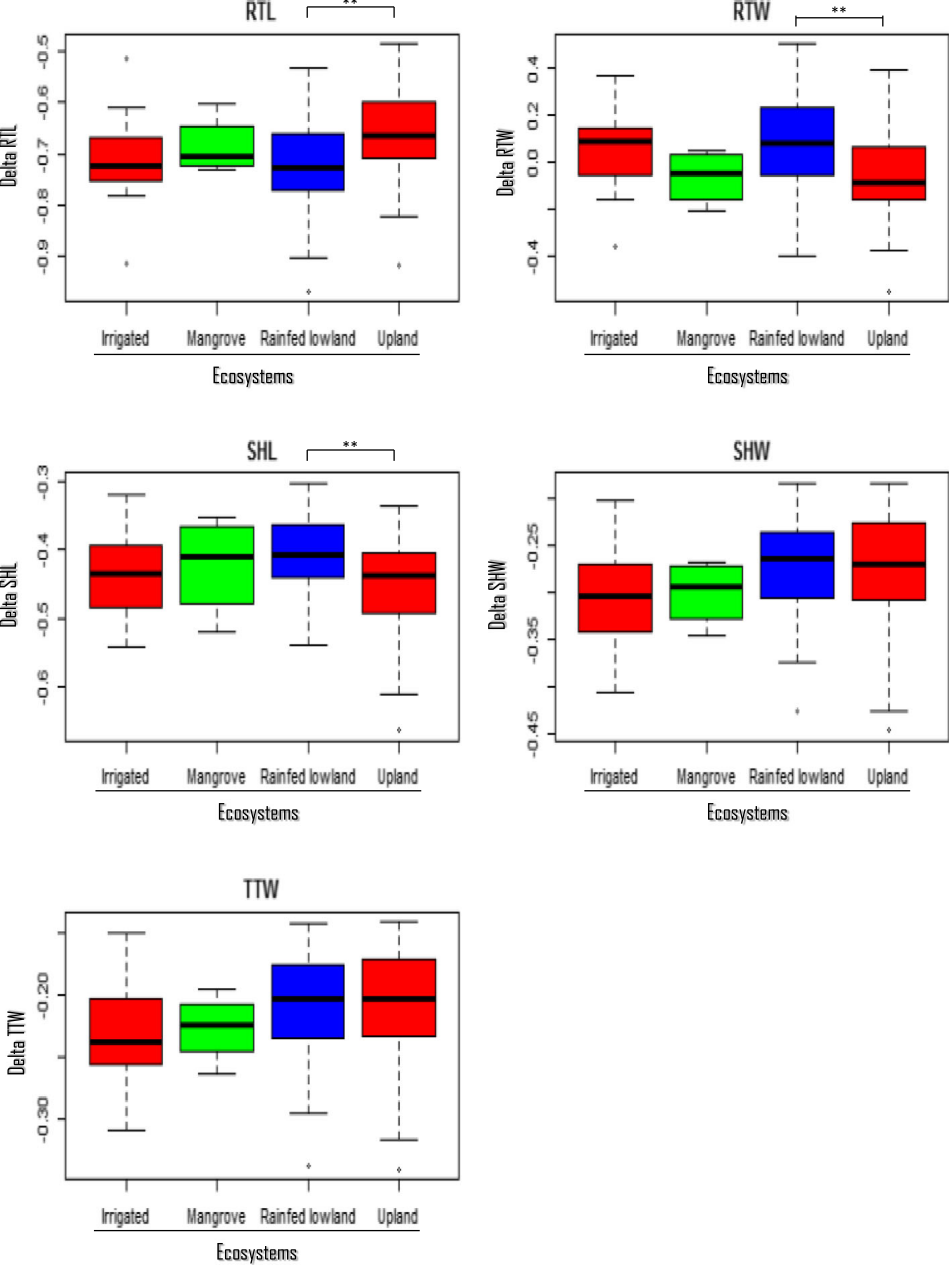
Phenotypic heterogeneity among ecosystems. The response to JA of rice accessions among four main ecosystems were compared. Student’s t- test was used to assess the differences between each couple of ecosystem. The (**) significant is shown with *p*- values < 0.01

The phenotypic heterogeneity among *Indica* and *Japonica* sub-groups (Fig. 3), among ecosystem (Fig. 4) and pair correlation of the 5 traits provide a descriptive overview of the data distribution for different rice subpopulations. In general, there are no significant differences within subgroups, and the effects of JA response for all parameters in 2 sub-groups are quite homogenous. This means that the response to JA in this study is a universal response for *O. sativa L.* and is not affected by sub-group population. Apparently, *Indica* accessions tend to have higher values for RTW and SHL reduction by JA compared to *Japonica* accessions, which have a slightly higher value on RTL, SHW and TTW (Fig. 3). The correlation between the 5 studied traits was also evaluated and shown in the Fig. 3. There is a very strong correlation in term of inhibition effect between SHW, SHL and TTW, especially for SHW and TTW with *r* value of 0.99. Interestingly, there are a weak correlation in term of inhibition effect of JA among root traits; and almost no correlation between RTW and RTL, which was indicated by the *r*^2^ value close to 0 (Fig. 3). Regards to their ecosytems, there are almost no clear differences between rice varieties which are Irrigated, Mangrove, and Rainfed lowland groups in term of response to JA treatment except there are significant differences between Rainfed lowland groups and Upland rice groups for RTL, SHL and RTW in response to JA treatment (*p*-value < 0.01) (Fig. 4).

### Association Mapping on Growth Traits in Response to JA Treatment

**Fig. 5:**
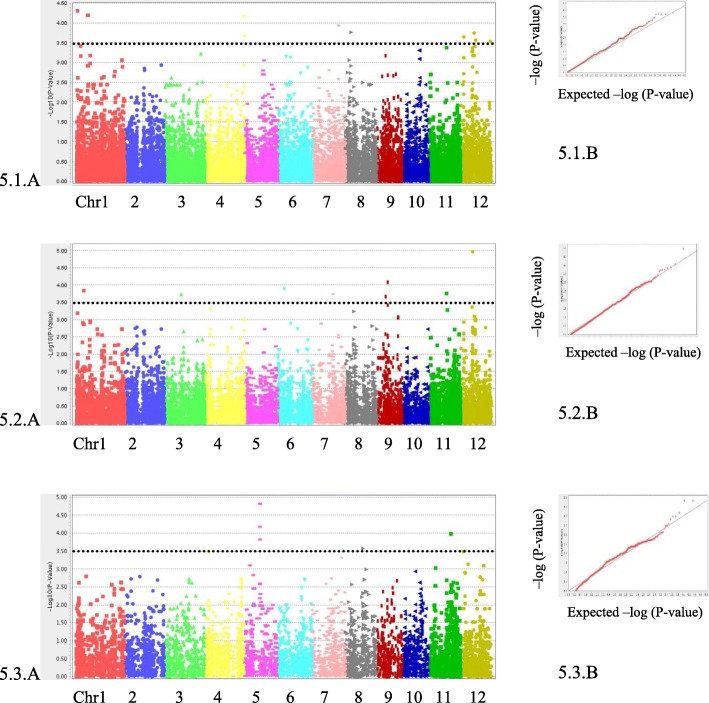
GWAS for the effects of exogenous JA on SHL. Manhattan plot (**a**) and Quantile-quantile plot (**b**) for shoot length in a whole (5.1) panel or *Indica* (5.2) or *Japonica* (5.3) sub-panel. The black line indicates the suggestive significance threshold, *p* = 3.0E-04

**Fig. 6:**
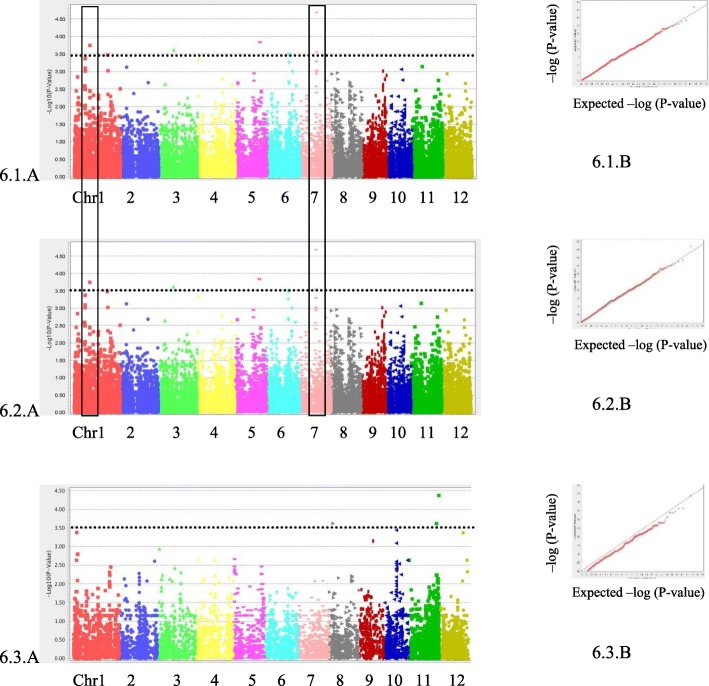
GWAS for the effects of exogenous JA on RTW. Manhattan plot (**a**) and Quantile-quantile plot (**b**) for root weight in a whole (6.1) panel or *Indica* (6.2) or *Japonica* (6.3) subpanel. The black line indicates the suggestive significance threshold, *p* = 3.0E-04

To explore whether specific SNPs markers in the genome are associated with the effects of exogenous JA on the phenotypic trait of rice growth, we performed GWAS on RTL, SHL, RTW, SHW and TTW using TASSEL v5.0. The rice collection has a bipolar structure with two main sub-groups, *Indica* and *Japonica* (Phung et al. 2014). Since GWAS results are greatly affected by the structure of the studied population, a GWAS was additionally performed with each sub-group to maintain a better population structure control and reduce the false positive rate. Therefore, the association mapping was examined for 155 accessions as whole panels, then separately, examining each independent sub-population including the *Indica* subpanel (95 accessions) and the *Japonica* sub-panel (60 accessions) (Figs. 5 and 6 and Additional file 4).

For all 5 traits in the whole panel, we applied the Mix Linear Model (MLM) that reconsiders both the population structure and kinship matrix. The results of genome-wide association mappings for effects of exogenous JA on SHL and RTW traits are illustrated in Figs. 5 and 6, respectively. Association mapping results for RTL, SHW and TTW traits are presented in the Additional file 4: Figures S4, S5 and S6, respectively. In the sub-group panel, especially for a small size panel such as *Japonica* (60 accessions), the General Linear Model (GLM) was also used in conjunction with the MLM, and Quantile-quantile (Q-Q) plots showed a good fit between observed *p*-values and theoretical *p*-values. In this study, most Q-Q plots display good accumulative distributions of observed *p*-values, which were well fitted with the expected uniform distribution for the smallest –log_10_(*p*-values).

Regarding the association of the effect of JA on SHL (Fig. 5), the Q-Q plot of all these plots for 3 panels were well fitted between observed *p*-values, and theoretical *p*-values. The statistical data pointed out 27 significant markers including 12 in the Whole panel (Fig. 5.1a), 8 significant markers in the *Indica* subpanel (Fig. 5.2a), and 7 significant markers in the *Japonica* subpanel (Fig. 5.3a). Chromosome 1 harbored 4 markers and chromosome 12 carried 4 markers for the whole panel, while chromosome 5 held 4 markers each for the *Japonica* subpanel.

The association mapping results describing the effect of JA on RTW is presented in Fig. 6. Q-Q plots showed well fitted compared to the expected line for all 3 panels. Thirteen significant markers were obtained. Data from MLM reported 5 significant markers to be shared between the Whole panel and *Indica* sub-panel.

Concerning the results on the RTL trait, presented in Additional file 4: Figure S4, there were 14 significant markers found, in which the whole panel harbored 12 markers, the *Indica* sub-panel held 2 markers. There was only one common marker shared between the whole panel and the *Indica* sub-panel (Dj02_29022158F). Association mappings on SHW (Additional file 4: Figure S5) showed 21 significant markers, in which the whole panel holds 4 markers, the *Indica* sub-panel holds 9 markers and the *Japonica* sub-panel holds 8 markers. There were 5 significant markers appeared in chromosome 1 in *Indica* sub-panel. Twenty-three significant markers were obtained on TTW trait including 6 significant markers in Whole panel, 8 in *Indica* sub-panel and 9 in *Japonica* sub-panel (Additional file 4: Figure S6).

### QTLs and Significant Markers Identification in the Whole Panel, *Indica* and *Japonica* Sub-Panel

Based on the results computed by TASSEL, 98 significant markers were identified at the threshold *p*-value < 3.0E-04. The linkage disequilibrium of each significant marker with their nearby markers was calculated to evaluate the possibility that these markers are associated within a population. Based on the calculation of linkage disequilibrium between SNPs computed using LD heatmap package, the list of QTLs was generated and is presented in Additional file 5: Table S4. As shown in this Table 3 QTLs for the RTL trait, 3 QTLs for RTW, 7 QTLs for SHL, 7 QTLs for SHW and 8 QTLs for TTW were identified and associated with the most significant markers in this study. The mean size of these QTLs is around 290 kb, and the smallest QTL was qTTW1 measuring 50 kb in length and the largest QTL was qTTW4 with length of 1423 kb. In total, 28 QTLs were recorded with 98 considered significant markers for the effects of exogenous JA treatments on these 5 traits. There are 14 QTLs indicated in the Whole panel, 7 QTLs in *Indica* sub-panel and 4 QTLs in *Japonica* sub-panel.

TTW is a trait with a highest number of QTLs and significant markers, totaling 8 QTLs and 22 significant markers. Seven markers of the whole panel, 11 markers of the *Indica* subpanel and 6 markers of the *Japonica* subpanel were obtained. For this parameter, 2 commons markers were shared between the Whole panel and *Japonica* sub-panel which were Sj04_32796870R and Sj04_32851563F in qTTW5. The qTTW3 with length of 532 kb in chromosome 1 contains 4 significant markers for the *Indica* sub-panel. The qTTW4 was also the longest QTL (1.4 Mbp), but it contained only 3 significant markers.

For RTW, 9 markers for 3 QTLs were discovered after applying the LD heatmap. In addition, the Whole panel and *Indica* subpanel shared 9 markers together. The QTL named qRTW3, measuring 221 kb at chromosome 7, which holds 7 markers at *p*-values at least less than 5.1E-04.

Regarding the RTL trait, LD heatmaps were applied and the outcome was 3 QTLs with 5 significant markers. The whole panel contained 4 markers, doubled than the *Indica* which contained 2 markers. The *Japonica* sub-panel did not harbor any significant markers. There is only one significant marker that appeared in both the whole panel and *Indica* subpanel at chromosome 2 (Dj02_29022158F).

In the SHW trait, there were 7 QTLs and 14 significant markers. The *Indica* sub-panel had most significant markers, 6 markers were identified in the Whole panel; the *Japonica* sub-panel contained only 1 marker. qSHW1 of 532 kb was the longest QTL responsible for SHW, but only 3 significant markers were obtained.

The SHL trait held 7 QTLs and 16 significant markers including 9 markers for Whole panel, only 1 for *Indica* sub-panel and 6 for *Japonica* sub-panel.

The common QTLs across 5 studied traits were determined. Notably, we detected 3 pairs of overlap QTLs for 5 given traits, the qSHW1/qTTW3 located in chromosome 1 including 4 significant markers (Dj01_38614134F, Dj01_38614137R, Sj01_38847771R and Sj01_38997056R); the qSHW7/qTTW8 located in chromosome 12 including 2 significant markers (Dj12_24056700R and Sj12_24257096R); the qSHW6/qSHL6 located in chromosome 12 included 3 lead markers (Sj12_02947152R, Sj12_02947164F and Dj12_2975990R). In short, SHW and TTW shared 2 common QTLs, SHL shared 1 common QTLs with SHW.

These results are the preliminary screening for the QTL associated with the growth of rice under stress condition. Further analysis should be conducted to functionally characterize these QTLs. The valided QTLs could help to dissect the underlying molecular mechanism controlling the defense/development trade off in rice.

### Polymorphism Combination of Significant Markers Inside Some Selected QTLs

**Fig. 7:**
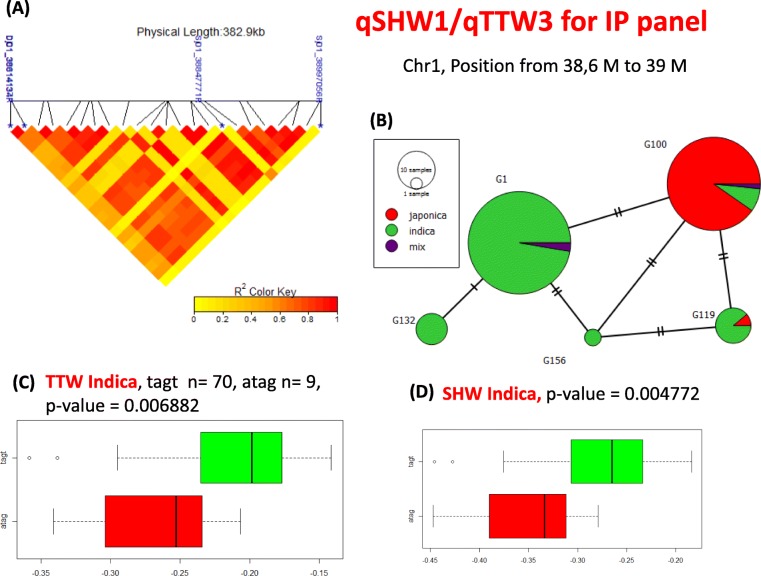
Haplotype analysis for qSHW1/qTTW3 **a** Linkage Disequilibrium heatmap in the peak region of association analysis GWAS for qSHW1/TTW3. Significant SNP indicated as blue star in the photo and the pattern pairwise r^2^ of the associated SNPs in the QTL indicated with color code. Red color means SNPs are strongly associated to each other and yellow color means no association. The Linkage Disequilibrium heat map was created using the “LDheatmap” package in R **b** Population architecture of accessions based on the allelic combination significant SNPs in each QTL. Population architecture image is created by PopArt1.7 software. **c** and **d** Effect of allelic combination of 2 main haplotypes of each QTL on the value of interested traits. Number of accessions for each haplotype is indicated as (n). Welch Two Sample t-test was used to assess the differences between two haplotypes. *, **, *** indicated significant difference at *p* value < 0.05, 0.01 and 0.001 respectively

**Fig. 8:**
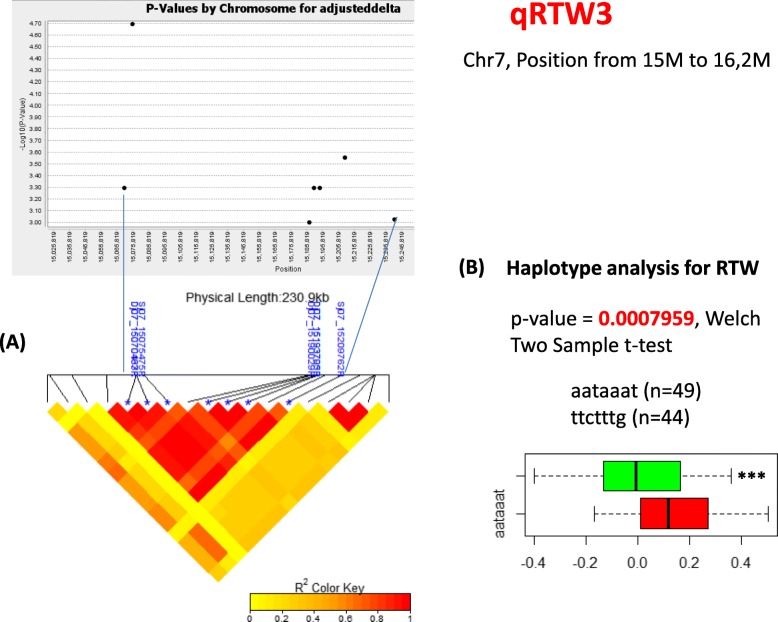
Haplotype analysis for qRTW3 **a** Linkage Disequilibrium heatmap in the peak region of association analysis GWAS for qRTW3. Significant SNP indicated as blue star in the photo and the pattern pairwise r^2^ of the associated SNPs in the QTL indicated with color code. Red color means SNPs are strongly associated to each other and yellow color means no association. The Linkage Disequilibrium heat map was created using the “LDheatmap” package in R. **b** Effect of allelic combination of 2 main haplotypes of each QTL on the value of interested traits. Number of accessions for each haplotype is indicated as (n). Welch Two Sample t-test was used to assess the differences between two haplotypes.*, **, *** indicated significant difference at *p* value < 0.05, 0.01 and 0.001 respectively

Using the significant SNPs located in each QTLs detected by GWAS (threshold *p*-value = 3.0 E-4) to make a haplotype sequence, polymorphism combination analysis were shown in the Figs. 7 and 8 and Additional file 6: Figure S7 for 4 selected QTLs with at least 4 markers. Related to the overlap QTL named qSHW1/TTW3 that was detected by GWAS for the *Indica* subgroup (Fig. 7), the effect of JA stress is more important with accessions contained the ATAG haplotype than those contained the TAGT haplotype in the QTLs to the SHW (*p*-value = 0.006882) and TTW (*p*-value = 0.004772). For qRTW3, all the 6 markers were associated with RTW by presenting a strong linkage in a red block showed in the Fig. 8a and revealed 2 major haplotypes in Fig. 8b. The positive effect of JA on RTW were associated with AATAAAT haplotype and significantly higher than those contained the TTCTTTG (*p*-value = 0.0007959) (Fig. 8c). For the qTTW5 in *Japonica* panel that was detected after the GWAS analysis (Additional file 6: Figure S7a), 2 main haplotypes TAGT and CGAG were revealed (Additional file 6: Figure S7b), and haplotype 1 has higher value compare to haplotype 2 (*p*-value = 0.002629) (Additional file 6: Figure S7c). These results support that the sequence variation in each QTL region can contribute to the phenotypic difference of interested traits.

### Identification of Candidate Genes Co-Localized with the Significant Markers

To identify candidate genes, annotations of the genes were first analyzed within the identified QTLs (25 kb up and downstream of the most significant SNP of the QTL) based on the MSU Rice Genome Annotation Project Database Release 7 (Kawahara et al. 2013). Among the significant markers found in the interval mapping of identified QTLs, there were 560 potential candidate genes found located on these QTLs with their annotation after removing all the transposons, hypothetical proteins and expressed proteins (Additional file 7: Table S5).

From this list, no known genes associated either with octadecanoid pathway (such as *OsAOS*, *OsAOC*, *OsJAR1*, etc.) or with jasmonate perception (such as *OsCOI1*, *OsJAZ* and *OsNINJA* genes) could be identified. We then conducted an annotation to functionally categorize the 560 genes using the second hierarchy level of the MapMan bins mapping. Detailed information and annotated function of these candidate genes is presented in Additional file 7: Table S5. The 10 most represented MapMan bins that represent 44% of the annotated genes indicated functions in regulation of DNA transcription, receptor kinases and calcium signaling, protein post-translational modification, protein degradation, biotic and abiotic stresses (Table 2).

**Table 2:**
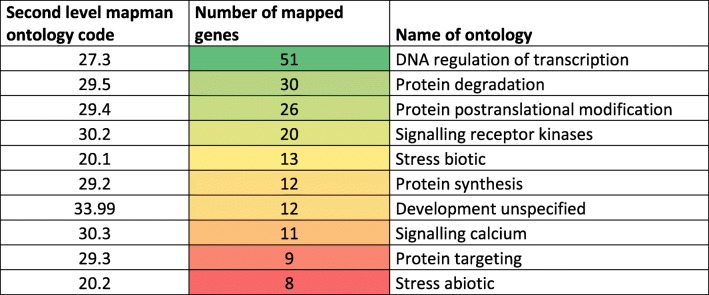
Repartition of MapMan mapping of the ten over-represented gene ontologies

### Identification of Candidate Genes Related to Jasmonate Signalling and Response in Shoot and Root

Exogenous application of jasmonate is known to lead in large changes in the transcriptome. In order to filter GWAS candidates, we integrated jasmonate RNAseq transcriptomes from the public TENOR database into the context of the GWAS analysis (Kawahara et al. 2016). We identified 232 genes showing up- and down-regulation under exogenous JA treatments with changes greater than 2-fold or lower − 2-fold during the time-course (6 times points from T0 to 24 h) with a FDR threshold set at 0,05. Among these genes, 137 and 95 are regulated by JA in root and shoot respectively (Additional file 8: Table S6). Interestingly, the annotated JA-responsive genes showed function mainly in regulation of DNA transcription (Additional file 9: Table S7). It was previously reported that transcription factors (TFs) play important roles in regulating gene expression responses to jasmonate (Pré et al. 2008). To explore which types of TF were present in JA regulated genes, we used the PlantTFDB to classify TFs (Jin et al. 2017). We identify 12 and 7 TF in root and shoot respectively to be differentially regulated by JA belonging to 7 TF families (Tables 3 and 4). In addition, 5 TFs were JA-responsive both in root and shoot transcriptomes. Among 14 differentially expressed TFs, 5 C2H2, 1 BHLH, 1 ERF, 1 NAC, and 1 LBD were found up-regulated. Altogether these data suggest that several key signalling elements involved in the jasmonate response are associated with the different QTLs identified in this study.

**Table 3:**
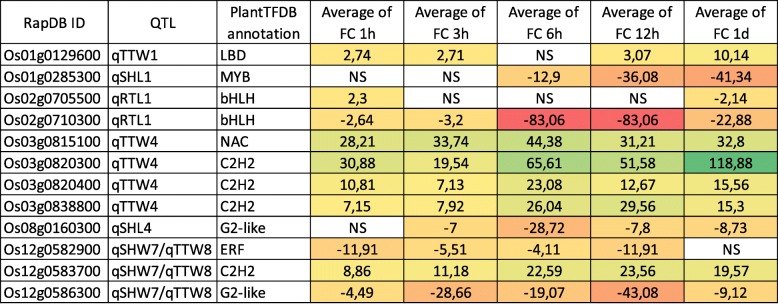
Expression pattern of rice transcription factors genes in response to jasmonate in root using RNAseq dataset from TENOR

*NS* Not significant, *FC* Fold change

**Table 4:**
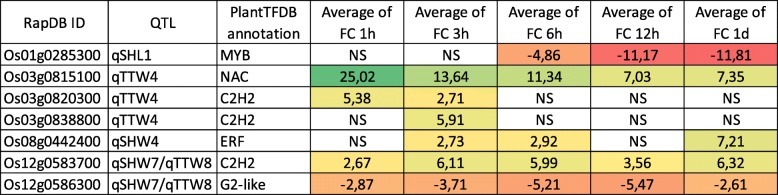
Expression pattern of rice transcription factors genes in response to jasmonate in shoot using RNAseq dataset from TENOR

*NS* Not significant, *FC* Fold change

## Discussion

### Natural Genetic Diversity of Rice Growth Inhibition in Response to Jasmonate

To better understand the role of JA in regulating complex growth response in rice and evaluate to which extent JA responses are modulated by genetic variability, we conducted a phenotyping analysis with 155 Vietnamese rice accessions based on a simple, robust and rapid phenotyping experiment in response to exogenous jasmonate. Jasmonate application has been widely used as a simple way to induce different types of defense response in plants, in general, and on rice, in particular. For example, Nahar and colleagues demonstrated that foliar application of exogenous JA triggered the systemic defense against the Root Knot Nematode (RKN) *M. graminicola* in rice root (Nahar et al. 2011). In addition, exogenous treatment of JA induces PR genes such as *OsPR1a* and *OsPR1b* and protect the rice root from the RKN infection (Nahar et al. 2011, 2012). In this study, we applied 5 μM of JA on plantlets growing in vitro during 7 days to score the effect on shoot and root growth. Seventeen plantlets per condition (with or without JA) for each genotype have been phenotyped for a total of 3094 rice plants showing significant repeatability and robustness. The use of 17 seedlings replicates per condition has improved the accuracy of the trait value (data not shown). Indeed, all traits showed a high heritability in our culture condition set up (Table 1). Recently, hormones treatments with auxin, cytokinin and abscisic acid have already been used to evaluate natural variation that shapes root system responses in *Arabidopsis* (Ristova et al. 2018). As we observed from our root phenotyping experiments in response to JA, they identify common response patterns relating to particular hormone perturbation modulating by specific genotypes (this study Fig. 2 and (Ristova et al. 2018)). While many major jasmonate signaling elements have been identified as genes responsible for the jasmonate-related short-root phenotype in few *Arabidopsis* accessions, our results based on the haplotype analysis open the possibility to use unexplored rice accessions with contrasted phenotypes as parent lines to identify new alleles that control JA pathway involved in growth inhibition processes.

### GWAS and Candidate Genes

While many studies have analyzed jasmonate signaling and response, to the best of our knowledge no study has explored JA-dependent genetic mechanisms involved in these molecular processes taking into account natural genetic variation. In this study, we applied the GWAS approach to identify QTLs associated with plant growth modulation by JA treatment in an *Oryza sativa* rice collection. As a result, 28 significant associations, including 3 QTLs for RTL, 3 QTLs for RTW, 7 QTLs for SHL, 7 QTLs for SHW and 8 QTLs for TTW. The non-compressed MLM models, with an option of re-estimating the markers we used in this study, are well fitted and appropriate for analysis, as shown by the low rate of false positives in the LD linkage calculation we observed. Many common significant associations with high-log (*p*-value) were found for both the whole panel and *Indica* panel, such as qSHL1, qRLT1, qTTW5, and qSHL3 (Additional file 5: Table S4). In addition, some QTLs were only found in the whole panel or in each sub-panel. It is likely that the limited size of the subpopulation, especially for the *Japonica* subpanel, which has only 55 accessions in the panel, could cause the decrease in the performance of GWAS analysis. Another hypothesis was already discussed in the study conducted by Phung et al in 2016. When dividing the panel into 2 subpanels, the number of polymorphic markers also decreased from 21,623 markers in the whole panel to 13,814 and 8821 markers for *Indica* and *Japonica*, respectively (Phung et al. 2016).

The GWAS analysis detected 28 QTLs with many genes annotated in the 25-kb window around a significant SNP. Based on MSU, RAPDB and MapMan annotation we were not able to identify genes already implicated in JA perception, biosynthesis and signalling. This observation could be explained by the long JA treatment we used to measure the growth parameter. Many JA biosynthesis genes as well as JAZs and TFs are known to play key function in the very early response and often play major roles where mutations can be detrimental in the wild. Another explanation could involve the presence of false negatives that are not identified by GWAS analysis.

Using available JA transcriptome we filtrated the 560 genes colocalized with the significant markers and found 42% of the candidate genes to be responsive to jasmonate, indicating that these genes are potential new players in jasmonate growth inhibition response pathway. Among this short JA-responsive gene list, we identified one Calmodulin-dependent protein kinases: OsSnRK1β, encoded by *Os03g17980* located in Chromosome 3. In plants, energy depletion caused by stress is sensed and coordinated by an energy-sensing protein kinase, named sucrose non-fermenting-1-related protein kinase-1 (SnRK1). In a study conducted by Filipe et al. (2018), the overexpression of *OsSnRK1α* confers broad-spectrum resistance in rice against various rice pathogens, including both hemibiotrophs (*Xoo PXO99, P. oryzae VT5M1*) and necrotrophs (*C. miyabeanus Cm988*, *R. solani AG1-1A* strain 16), while inhibiting the growth and development of rice. Interestingly, OsSnRK1α activated the JA signaling and response pathway by boosting the expressions of a JA biosynthesis gene (*OsAOS2*) and JA-related genes such as *JIOsPR10* and *OsJAMyb* after inoculating the plant with *Pyricularia oryzae* (Filipe et al. 2018)*.* Consequently, Filipe suggested that *OsSnRK1α* plays a key role in the energy balance between plant defense and plant development (Filipe et al. 2018). Such a role should be researched for *OsSnRK1β*, which we identified in a QTL associated with RTL inhibition.

In addition, other candidate genes that were found have a function related to secondary metabolism. For example, the 2 genes *Os01g18110* and *Os01g18120*, located in Chromosome 1, near the Sj01_10048970F marker, were annotated as Cinnamoyl CoA reductase (*OsCCR4* and *OsCCR5* respectively), which are the first responsible enzymes specific to the pathway for lignin biosynthesis (Park et al. 2017). In *Cassia tora*, application of methyl jasmonate at 10 μM promoted root sensitivity to aluminum-induced apoplastic peroxidase activity and H_2_O_2_ and lignin accumulation (Xue et al. 2008). In 2015, while studying the Scorpion peptide LqhIT2 in rice transgenic plants, Tianpei et al. (2015) found that the pathways downstream of LOX, such as the JA pathway, were activated and then lignin content was increased accordingly. They suggested that JA induce expression of genes involved in the phenylpropanoid pathway, leading to lignin accumulation in rice tissues (Taheri and Tarighi 2010). In 2011, while studying the induction of lignin biosynthesis induced by cell wall damage in *Arabidopsis*, Denness and his colleagues found that the plant regulates the biosynthesis of lignin through the interaction between the JA-dependent process and reactive oxygen species (Denness et al. 2011). A study on RICE SALT SENSITIVE3 protein encoded by *RSS3*- shown that this gene regulates root cell elongation under salinity condition (Toda et al. 2013). RSS3-JAZ-bHLH complex regulated jasmonate-induced gene expression involved in the cell wall metabolism including lignin and phenyl- propanoids biosynthesis (Toda et al. 2013). *OsCCR4* and *OsCCR5* were found in relation to two root traits: the root mass and the RTL. Research using RiceXpro showed that *OsCCR5* was up-regulated after 6 h when roots of 7 day-old rice plantlets were treated with 100 μM of JA. Consistent with the prediction of molecular process, this finding suggests that the effect of JA enhances the biosynthesis of some secondary macromolecules in roots such as lignin, making rice roots more resistant to adverse conditions. Indeed, we observed that the roots were not only shorter in response to JA but also less flexible compared to the untreated roots (data not shown). Diversity of root cell wall modification in response to JA will be explored further by metabolomics phenotyping of the rice collection.

The MapMan annotation of the candidate genes allowed to highlight a strong representation of genes involved in the regulation of DNA transcription and signaling. The search for JA-responsive transcription factor via TFDB led to the identification of 12 TFs. Among them, several studies emphasized their regulation in response to stress like drought (*Os02g0705500,* (Minh-Thu et al. 2018)), excessive Fe in roots (*Os01g0129600*, (Bashir et al. 2014)), macronutrient deficiency (*Os12g0582900*, (Takehisa et al. 2015)), high temperature (*Os08g0442400*, (Endo et al. 2009)) and infections by *Xanthomonas oryzae* pv. *oryzae* (*Os12g0586300,* (Wang et al. 2019); *Os03g0820400*, (Yi et al. 2014)).

Functional analyzes were also conducted for the TFs *SNAC1* and *ZFP252* belonging respectively to the families NAC and C2H2 *SNAC1* is induced in guard cells by drought and enhances drought resistance in overexpression transgenic rice in the field under drought salt stress conditions (Hu et al. 2006). For *ZFP252*, it was also reported that this TF increased tolerance to salt and drought stresses by regulating the content of proline and soluble sugars under salt and drought stress (Xu et al. 2008). Unlike many TFs involved in stress tolerance responses, overexpression of *SNAC1* or *ZFP252*, does not lead to growth inhibition or yield penalty of transgenic plants (Hu et al. 2006; Xu et al. 2008). Transcriptome data sets from TENOR show that these two TFs are not only induced by JA in shoot but also in roots. Therefore, it would be interesting to determine whether the expression of these genes is differentially regulated in the contrasting genotypes for the JA response and to identify in roots their function in response to stresses.

## Conclusion

The GWAS is a powerful tool to mine the valuable alleles hidden in the biodiversity richness of rice (Korte and Farlow 2013). This marks the first time that JA-related growth inhibition traits have been characterized in rice by GWAS. Identification of QTLs associated with the growth of rice treated with exogenous JA can help to dissect the underlying molecular mechanism controlling the defense/development trade off in rice. Further analysis should be conducted to functionally characterize these QTLs. We already develop a mapping segregating for four QTLs by generating a haplotype map. This analysis will be completed by using some accessions to re-sequencing at higher depth to confirm the results. Several promising candidate genes such as *OsSnRK1β* will be investigated for expression in contrasting accessions. The results of this research could help to create stress-tolerant rice accessions while maintaining plant growth.

## Materials and Methods

### Plant Materials

The collection used in the experiment was composed of 155 Vietnamese rice accessions whose seeds were provided by the Plant Resource Center in Hanoi, Vietnam. These accessions were mostly landrace lines collected from various regions and ecosystems in Vietnam (Phung et al. 2014). The accession IR64 was added as internal controls in the phenotyping experiment. The GBS methods and the marker selection process, as described by Phung et al. (2014), were used to generate the genotypic data. The genotypic data consisted of 21,623 markers with no missing data that could be used for genome-wide association purposes.

### Growth Condition

The rice collection was grown in vitro under axenic conditions in a programmable growth chamber (DAIHAN Scientific, Thermo Stable GC-450) under a 12 h daily light cycle at 80% humidity. The light intensity was 12,000 Lux. The temperature was adjusted to 26 °C and 28 °C during dark and light phases, respectively. Initially, the seeds were kept in the oven at 50 °C for 3 days to break dormancy. Then, they were decorticated manually and surface-sterilized by shaking with 70% ethanol for 2 min. After being rinsed with sterilized distilled water, the seeds were soaked in commercial bleach (3.8–4% sodium hypochlorite) with 2 drops of Tween 20 and were agitated for 25 min. Next, the seeds were washed 7–8 times with sterilized distilled water and left overnight in the dark at 26 °C to maximize water absorbance. Surface-sterilized seeds were sowed on an agar Petri dish at 6% w/v. After storing the Petri dish in a growth chamber for 1 day, each plantlet was transferred to a glass test tube with Murashige and Skoog (MS) medium at half concentration, pH = 5.8 (adjusted by potassium hydroxide), containing 2 g/L Phytagel (Sigma Aldrich), supplemented with or without jasmonic acid (TCI-Japan) at 5 μM for the phenotyping experiment. The tubes were subsequently transferred to a growth chamber, and the phenotyping process occurred 7 days after JA treatment.

### Phenotyping Experiment

The experiment was conducted using an augmented randomized complete block design, with 7 blocks, each with 26 accessions such that the IR64 control was allocated in each of the 7 blocks and 25 others landraces accessions were allocated only once in the design (Boyle and Montgomery 1996). The experiment was performed in triplicates by dividing the experiment into three sub-blocks. Each sub-block had at least 5 to 6 replicates per condition and per accession. In each sub-block, the position of each replicate was randomized using IRRISTAT 4.0 software to avoid systematic effects due to positioning. For each plant, the length of the longest root (RTL) and the length of the shoot (SHL) were measured. After drying at 55 °C in the oven for 1 week, the weight of the whole plant (TTW) and of the shoot portion (SHW) were measured. The RTW was calculated by subtracting SHW from TTW. To ensure the homogenous germination rate, accessions having a low germination rate or slow germination time were discarded from the study. In total, 155 rice accessions were assessed in this study.

### Statistical Analysis

To analyze the phenotype data of the 155 Vietnamese rice accessions, analysis of variance (ANOVA) was performed to verify the effects of various blocks, genotypes and JA treatment. The block effect was calculated based on the phenotype of the IR64 controls. As the block effect was significant, the data were normalized by using the ‘lme4’ package in R. Next, the broad-sense heritability coefficient *H*^*2*^ was calculated. Effects of JA on each trait were used as phenotype data in genome-wide association mapping. All statistical tests and analyses in studying the phenotype data of 155 Vietnamese rice accessions were run under R software version 3.4.3.

### Genome-Wide Association Mapping

Association analyses were conducted on the whole panel having 155 rice accessions and a collection of 21,632 markers using Tassel v5.0 (Bradbury et al. 2007) to identify genetic variants associated with responses to exogenous JA treatment. From genotype data, a kinship matrix was generated by centered Identity by State (IBS) method proposed by TASSEL. To consider the structure of the collection derived from a previous study by Phung et al. (2014), a principle component analysis (PCA) with the top 6 components was also calculated from haplotype data, and an eigenvalue decomposition of the covariance matrix was executed, as recommended by Reich et al. (2008). Both the Generalized Linear Model (GLM) and non-compressed Mixed Linear Model (MLM) were applied, but we mainly used MLM with an option of re-evaluation of variance components for each marker to process the kinship matrix and a matrix combining both phenotype data and structure of the panel, producing a GWAS result. Then, the quantile-quantile plot (Q-Q plot) and Manhattan plot were drawn using TASSEL. The q-value corresponds to adjusted *p*-value after FDR analysis was computed to estimate the false discovery rate using package “qvalue” in R (Phung et al. 2016). However, in order to make the comparison within populations and across traits, we applied a less stringent *p*-value of 3.0E-4 as suggestive threshold to declare that an association was significant.

### Linkage Disequilibrium and QTL Selection

Subsequent to achieving a list of significant markers, a linkage disequilibrium heatmap for each marker was generated to confirm if this marker belonged to a quantitative-trait locus (QTL). The “LD heatmap” package allowed us to calculate pairwise linkage disequilibria between markers and to visualize the results (Shin et al. 2006). Only regions having at least one significant marker associated with nearby markers were considered as a QTL.

### Screening for Annotated Genes and Transcriptome Analysis

The positions of found QTLs were then used to screen for candidate genes on the MSU Rice Genome Annotation Project Database, release 7.0 (Kawahara et al. 2013). Only expressed genes located around 25 kb before and after each significant marker were selected. A list of candidate genes with their annotation information was formulated.

For each of the 560 genes, we converted the MSU ID to RapDB ID using the RapDB ID converter (Sakai et al. 2013). These RapDB gene IDs allowed to map MapMan ontologies using the BinTree RAPDB-IRGSP1.0 version 1.0 (Thimm et al. 2004). Granularity of the bins was further reduced to the second level (e.g. 27.2: RNA.transcription) for readability. Also, RapDB gene IDs were used to import TF family annotation from PlantTFDB 4.0 mapping (Jin et al. 2017). In order to perform a cross analysis, the 560-gene list was used as a query into Tenor database (Kawahara et al. 2016) from which we pulled the FC and associated FDR values for all associated transcripts in response to JA in root and shoot. For each transcript and each time point, we retained the values presenting a FC </> -2/2 with FDR< 0.05. For each gene and each time point, we then calculated the average FC and standard deviation, thus providing a list of genes both detected in the 50 kb interval of the QTLs of interest, and strongly and significantly differentially expressed in response to JA in root and/or shoot.

### Haplotype Analysis

The significant markers of each LD block of interested QTL were used to define haplotype using alignment Nexus file. The 2 main haplotype was selected based on the geographic maps created by PopArt software version 1.7 (Population Analysis with Reticulate Trees software) (http://popart.otago.ac.nz) then we compare with the phenotype of each haplotype in order to confirm if the sequence variation in each QTL region can contribute to the phenotypic difference of interested traits.

## Additional Files

Additional file 1: **Figures S1**, **S2**, **S3** and **Table S1.** Growth inhibition of 10 representative accessions in response to JA. **Figure S1.** for histogram of distribution of each trait. **Figure S2.** presents the variation of 5 traits between 10 representative’s accessions in non-treated and 5 μM JA treated condition. **Figure S3.** illustrated the percentage reduction of each trait after JA treatment compare to the non-treatment. **Table S1.** expressed the heritability coefficient of each traits. (DOCX 969 kb)

Additional file 2: **Table S2.** List of 155 Vietnamese rice accessions used in this study with information on their gene bank number, their sub-populations groups as well as their ecosystems. (DOCX 52 kb)

Additional file 3: **Table S3.** Effects of 5 μM JA on the phenotypic variation of the growth traits for 155 rice accessions. (CSV 12 kb)

Additional file 4: **Figures S4**, **S5** and **S6.** GWAS for the effects of exogenous JA on RTL, SHW and TTW. Manhattan plot (A) and Quantile-quantile plot (B) for RTL (**Figure S4**), SHW (**Figure S5**) and TTW (**Figure S6**) in a whole (S.x.1) panel or *Indica* (S.x.2) or *Japonica* (S.x.3) subpanel. The blue line indicates the suggestive significance threshold, *p* = 3.0E-04. Black rectangle represent common significant SNPs within panels. (DOCX 1060 kb)

Additional file 5: **Table S4.** List of signification markers of detected QTLs at *p*-value < 3.0E-04. The start (site 1) and end positions (site 2) of each QTL were estimated by expanded 25 kb to both terminals base on the LD linkage analysis. Chr for chromosome, RTW for root dry weight, SHW for shoot dry weight, RTL for root length, SHL for shoot length. (XLSX 24 kb)

Additional file 6: **Figure S7.** Haplotype analysis for qTTW5. (A) Linkage Disequilibrium heatmap in the peak region of association analysis GWAS for qRTW3. Significant SNP indicated as blue star in the photo and the pattern pairwise r2 of the associated SNPs in the QTL indicated with color code. Red color means SNPs are strongly associated to each other and yellow color means no association. The Linkage Disequilibrium heat map was created using the “LDheatmap” package in R (B) Population architecture of accessions based on the allelic combination significant SNPs in each QTL. Population architechture image is created by PopArt1.7 software . (C) Effect of allelic combination of 2 main haplotypes of each QTL on the value of interested traits. Number of accessions for each haplotype is indicated as (n). Welch Two Sample t-test was used to assess the differences between two haplotypes.*, **, *** indicated significant difference at *p* value < 0.05, 0.01 and 0.001 respectively. (PPTX 116 kb)

Additional file 7: **Table S5.** List of candidate genes found ±25 kb around the significant markers and MapMan and TFDB annotations. (XLSX 2416 kb)

Additional file 8: **Table S6.** Expression pattern of candidate genes in response to jasmonate in shoot and root using RNAseq dataset from TENOR. (XLSX 20 kb)

Additional file 9: **Table S7.** MapMan annotation of the JA-responsive candidate genes identified in the TENOR transcriptome database. (XLSX 384 kb)

## Data Availability

The data sets supporting the results of this article are included within the article and its supporting files.
