# Supplementary material for: Unraveling the Genetic Elements Involved in Shoot and Root Growth Regulation by Jasmonate in Rice Using a Genome-Wide Association Study
Source: Rice (N Y). 2019 Sep 4;12:69. doi: 10.1186/s12284-019-0327-5 (PMC6726733; doi:10.1186/s12284-019-0327-5)
Supplement: Supplementary file 1 — Figures S1, S2, S3 and Table S1. Growth inhibition of 10 representative accessions in response to JA. Figure S1. for histogram of distribution of each trait. Figure S2. presents the variation of 5 traits between 10 representative’s accessions in non-treated and 5 μM JA treated condition. Figure S3. illustrated the percentage reduction of each trait after JA treatment compare to the non-treatment. Table S1. expressed the heritability coefficient of each traits. (DOCX 969 kb) [file 12284_2019_327_MOESM1_ESM.docx]

**1. Histogram of distribution of each trait:**


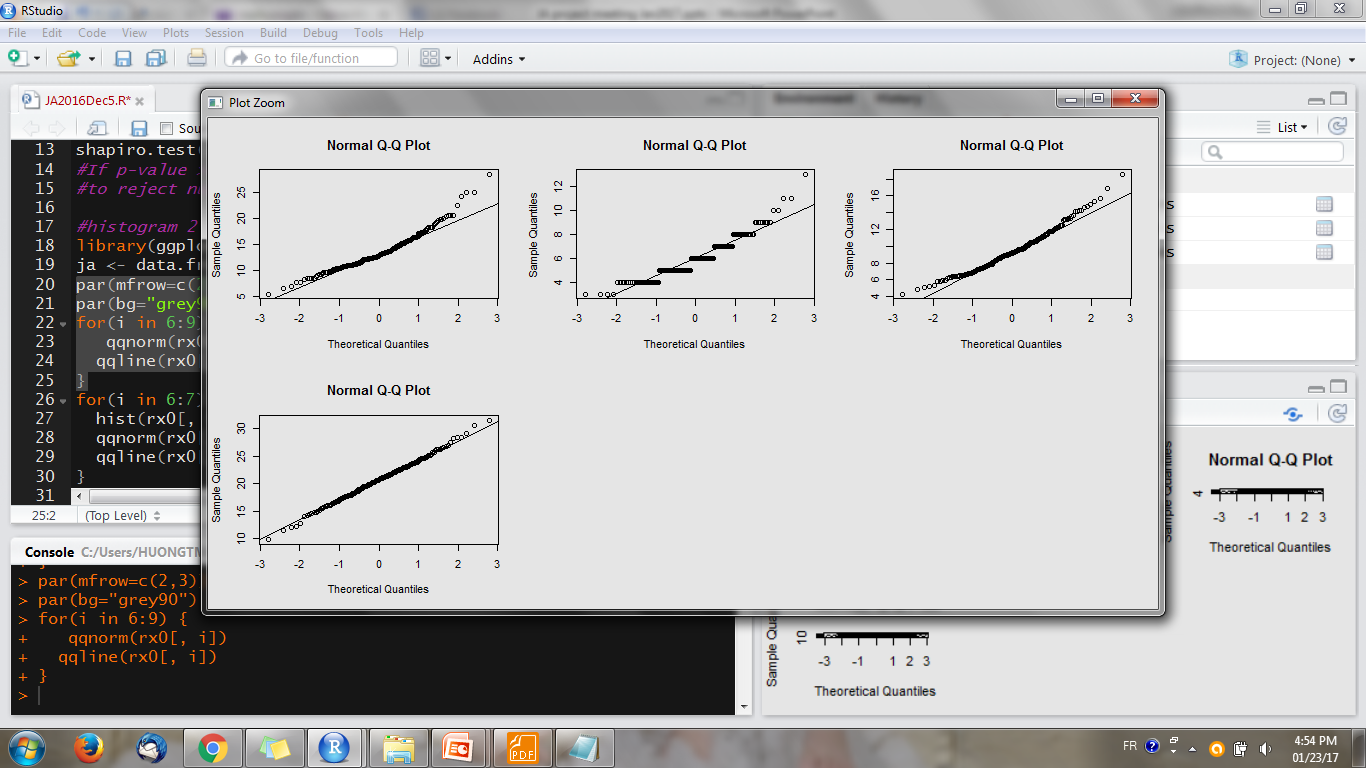

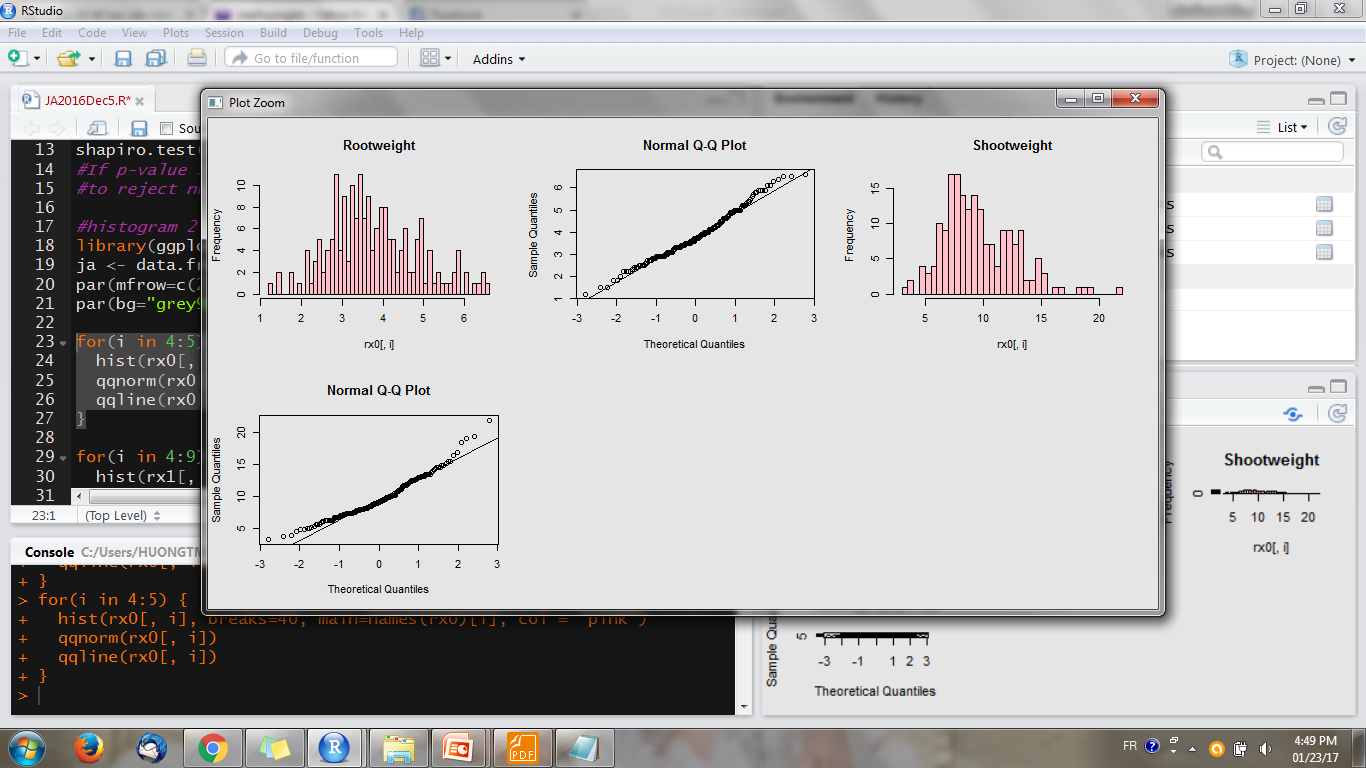

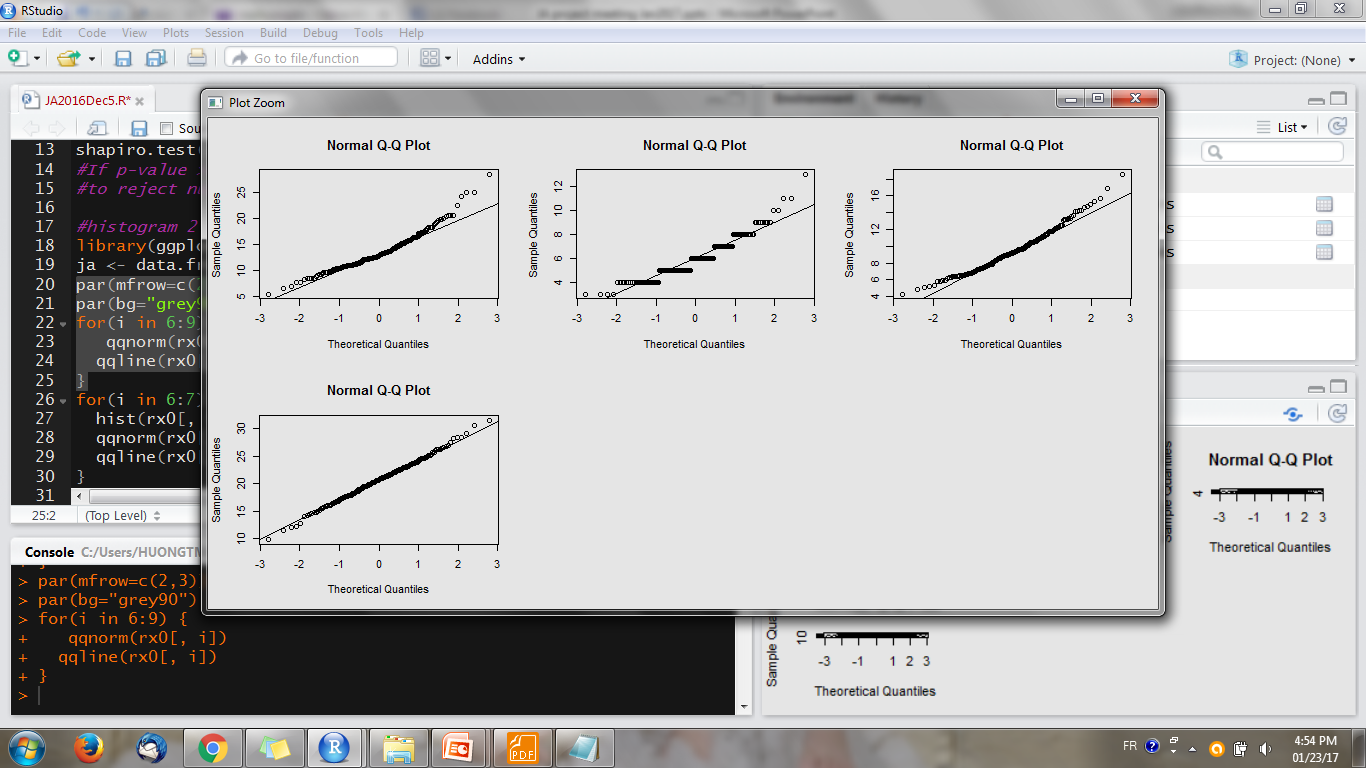

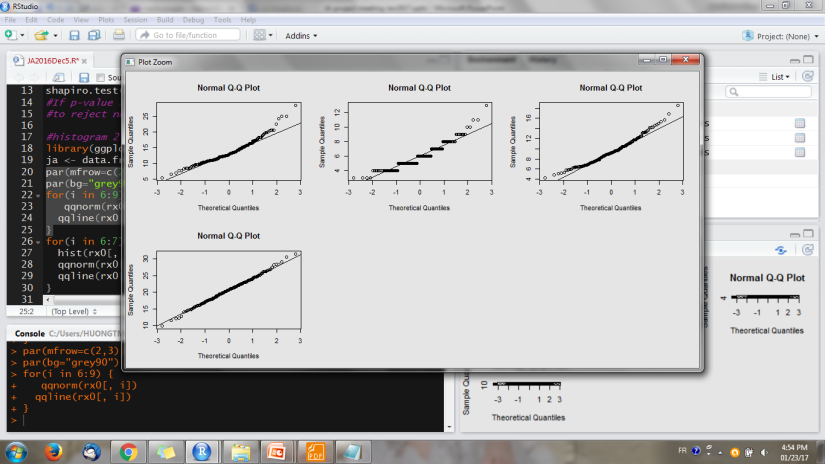

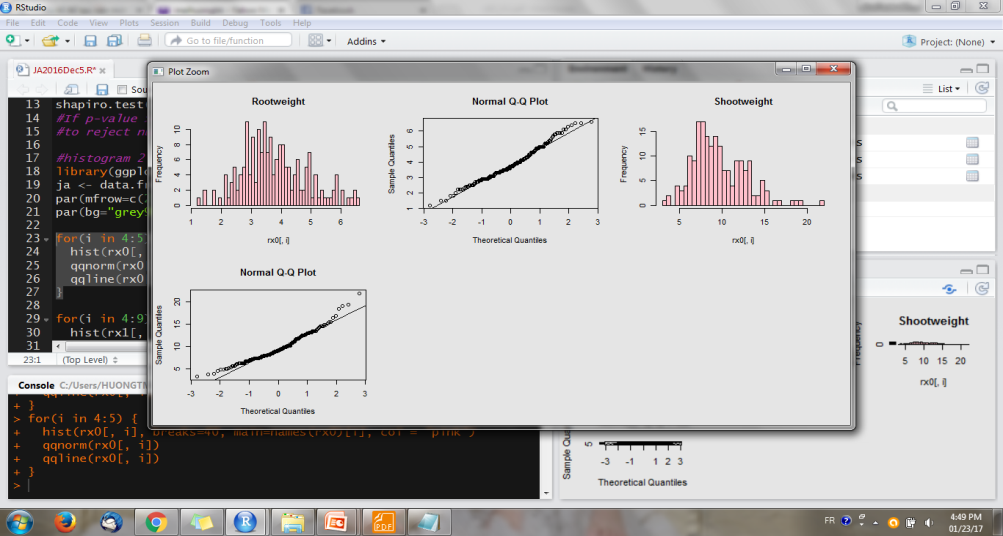

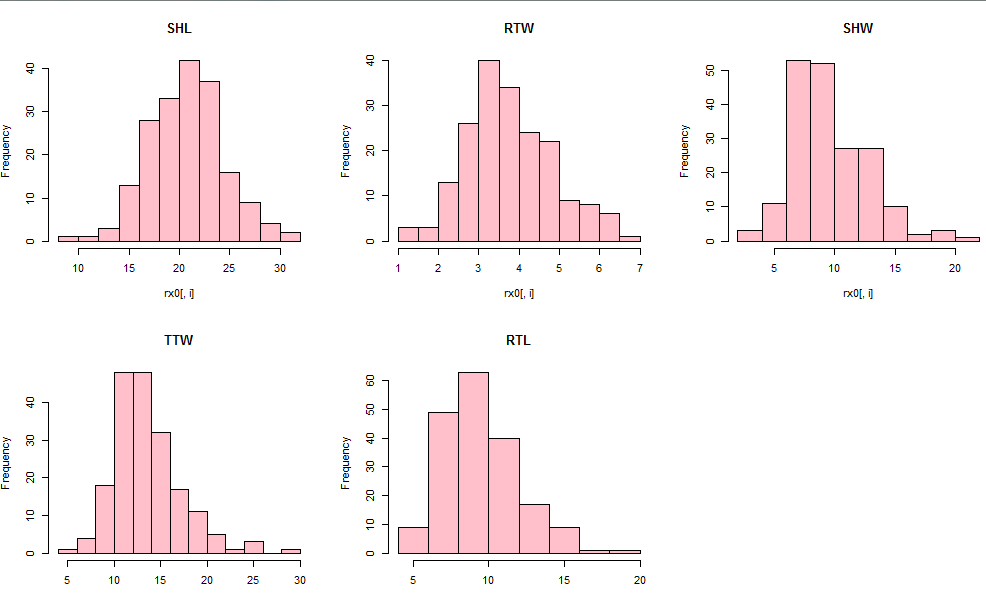


Figure S1a. Non-treated JA (J0) Five parameters were collected from 7 day-old plantlets growth in 1/2 MS without JA. Data represent means of 20 plantlets


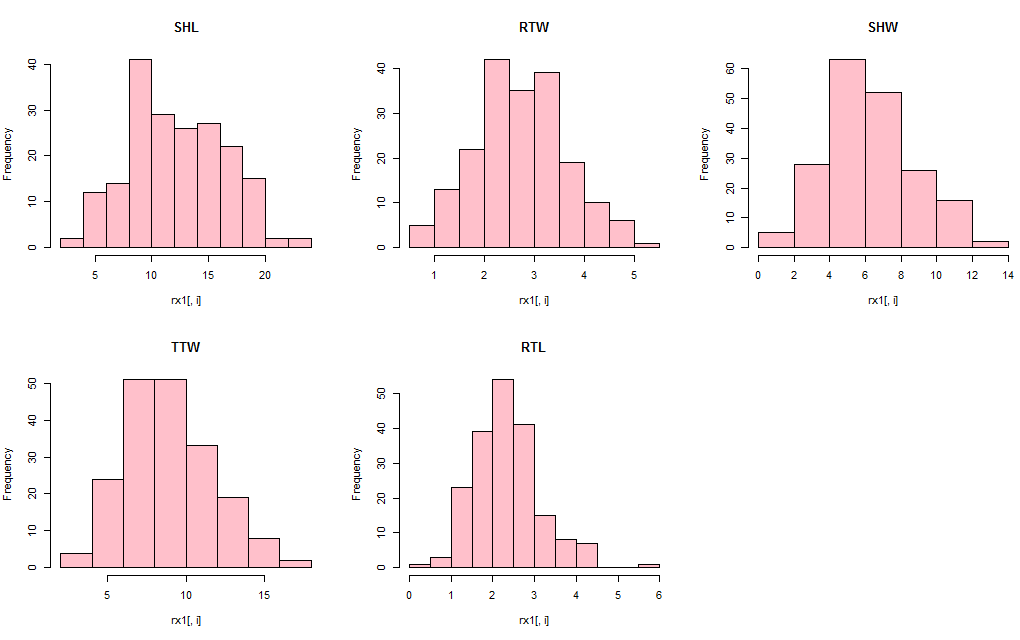


Figure S1b. Treated JA (J1) Five parameters were collected from 7 day-old plantlets growth in 1/2 MS with 5µM JA. Data represent means of 20 plantlets

**2. Variation of 5 traits between 10 representative’s accessions in non treated and 5µ JA treated condition**


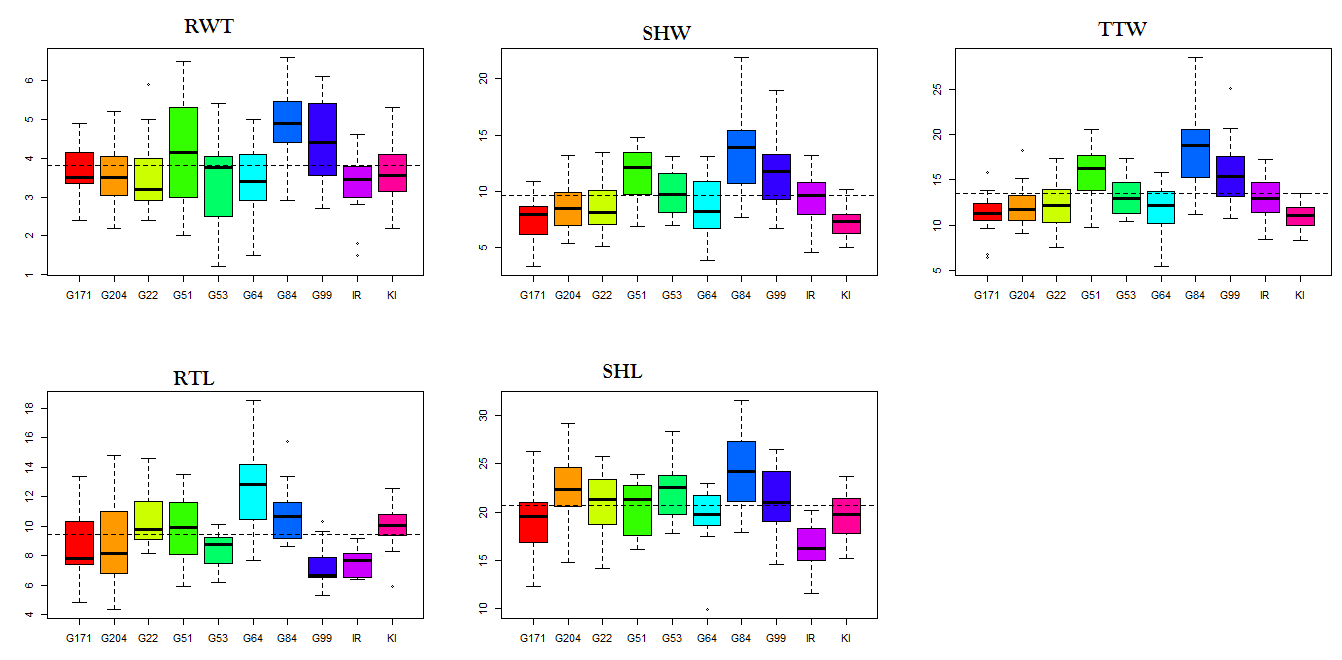


Figure S2a. Non-treated JA (J0) Five parameters were collected from 7 day-old plantlets growth in 1/2 MS without JA. Data represent means of 20 plantlets


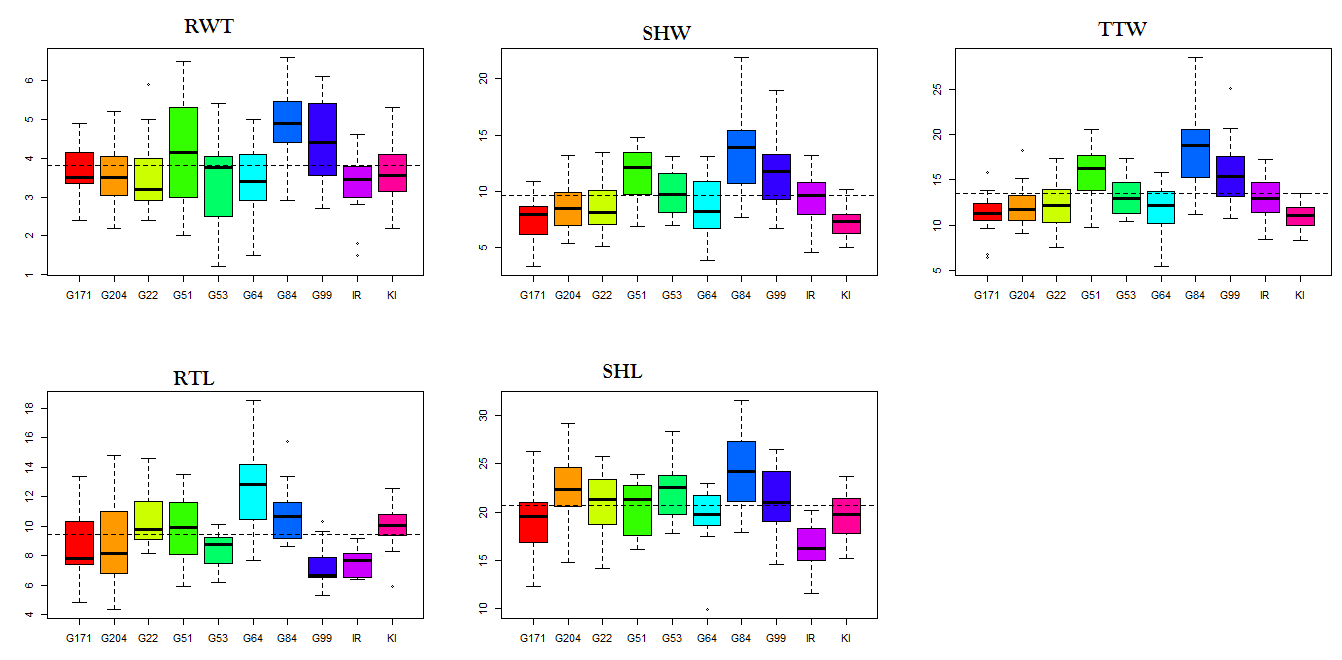


Figure S2b. Treated JA (J1) Five parameters were collected from 7 day-old plantlets growth in 1/2 MS with 5µM JA. Data represent means of 20 plantlets

**3. Percentage reduction of each trait after JA treatment**

Figure S3. Percentage reduction of each trait after JA treatment including root length (RTL), shoot length (SHL), root weight (RWT), shoot weight (SHW) and total weight (TTW) was calculated by offset of means of treated trait to means of non-treated trait then dividing by means of non-treated trait.

**4. The heritability coefficient**

| **Traits** | **RWT** | **SHW** | **TTW** | **RTL** | **SHL** |
| --- | --- | --- | --- | --- | --- |
| Heritability coefficient | 0.9 | 0.95 | 0.96 | 0.94 | 0.91 |

Table S1. Heritability coefficient of each traits
