# Supplementary material for: Unraveling the Genetic Elements Involved in Shoot and Root Growth Regulation by Jasmonate in Rice Using a Genome-Wide Association Study
Source: Rice (N Y). 2019 Sep 4;12:69. doi: 10.1186/s12284-019-0327-5 (PMC6726733; doi:10.1186/s12284-019-0327-5)
Supplement: Supplementary file 4 — Figures S4, S5 and S6. GWAS for the effects of exogenous JA on RTL, SHW and TTW. Manhattan plot (A) and Quantile-quantile plot (B) for RTL (Figure S4), SHW (Figure S5) and TTW (Figure S6) in a whole (S.x.1) panel or Indica (S.x.2) or Japonica (S.x.3) subpanel. The blue line indicates the suggestive significance threshold, p = 3.0E-04. Black rectangle represent common significant SNPs within panels. (DOCX 1060 kb) [file 12284_2019_327_MOESM4_ESM.docx]

| Add 4. Fig 4.1.A **P-values by Chromosome for RTL**  –log (P-value)  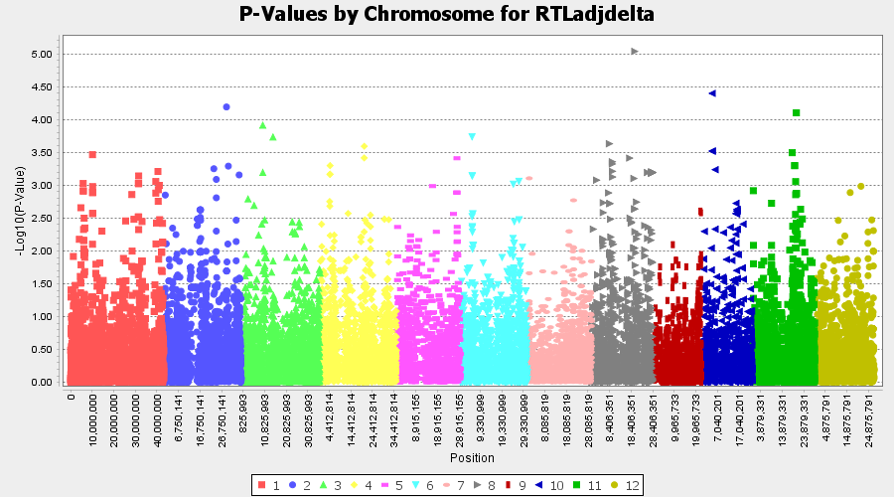  Expected –log (P-value)  Chr1 2 3 4 5 6 7 8 9 10 11 12 | Add 4. Fig 4.1.B 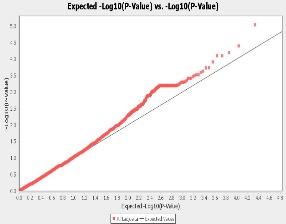 |
| --- | --- |
| Add 4. Fig 4.2.A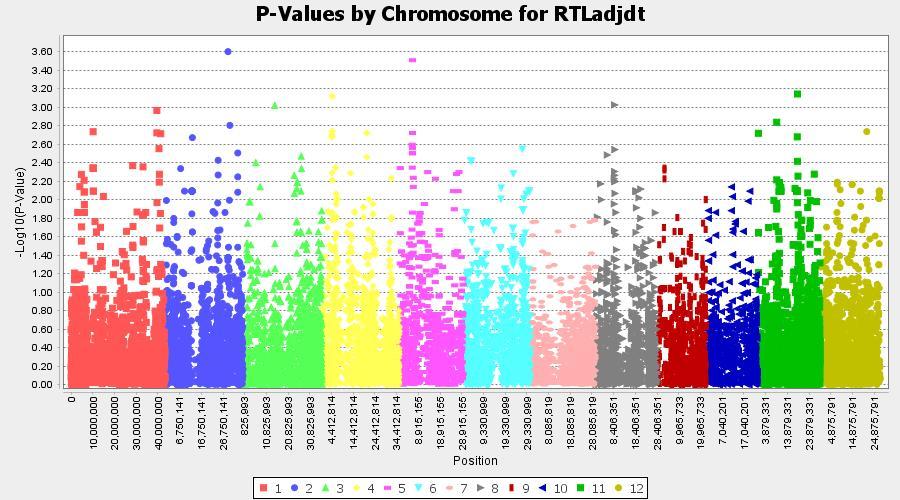  Expected –log (P-value)  –log (P-value)  Chr1 2 3 4 5 6 7 8 9 10 11 12 | Add 4. Fig 4.2.B 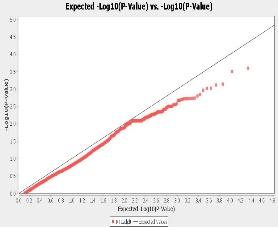 |
| Add 4. Fig 4.3.A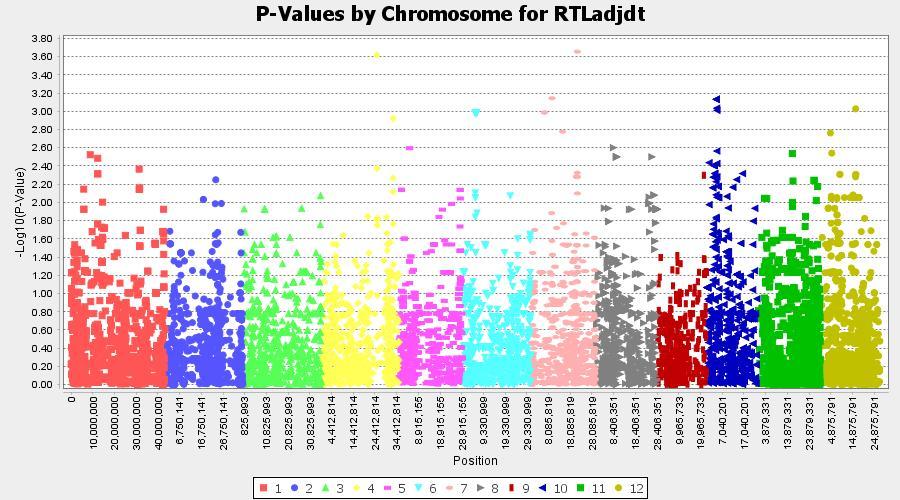  Expected –log (P-value)  –log (P-value)  Chr1 2 3 4 5 6 7 8 9 10 11 12 | Add 4. Fig 4.3.B 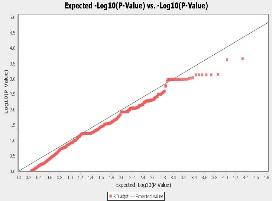 |

**Additional file 4. Fig S4: GWAS on RTL.**

| Add 4. Fig 5.1.A **P-values by Chromosome for SHW** 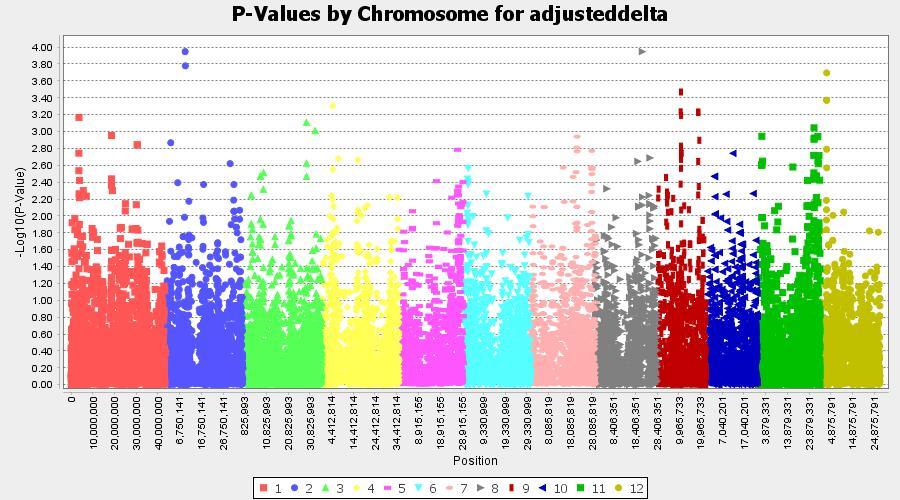  Expected –log (P-value)  –log (P-value)  Chr1 2 3 4 5 6 7 8 9 10 11 12 | Add 4. Fig 5.1.B 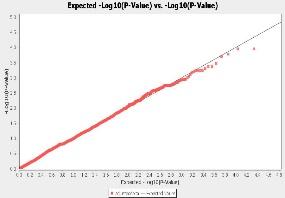 | |
| --- | --- | --- |
| Add 4. Fig 5.2.A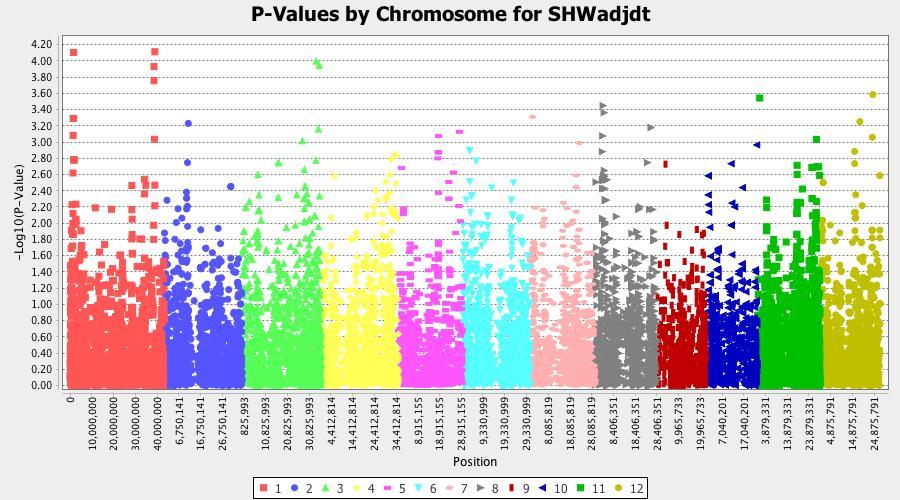  –log (P-value)  Chr1 2 3 4 5 6 7 8 9 10 11 12 | Add 4. Fig 5.2.B 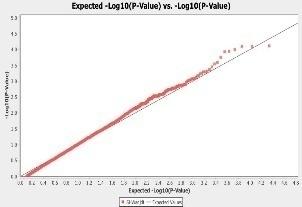  Expected –log (P-value) | |
| Add 4. Fig 5.3.A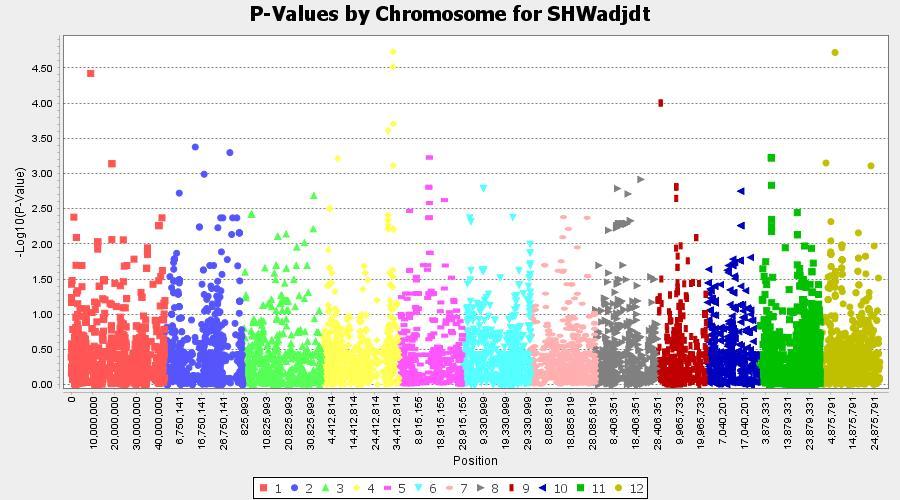  –log (P-value)  Chr1 2 3 4 5 6 7 8 9 10 11 12  **Additional file 4. Fig 5: GWAS on SHW** | Add 4. Fig 5.3.B 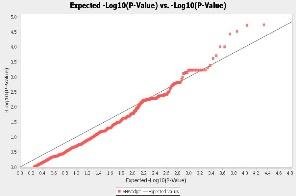  Expected –log (P-value) | |
| Add 4. Fig 6.1.A **P-values by Chromosome for TTW** 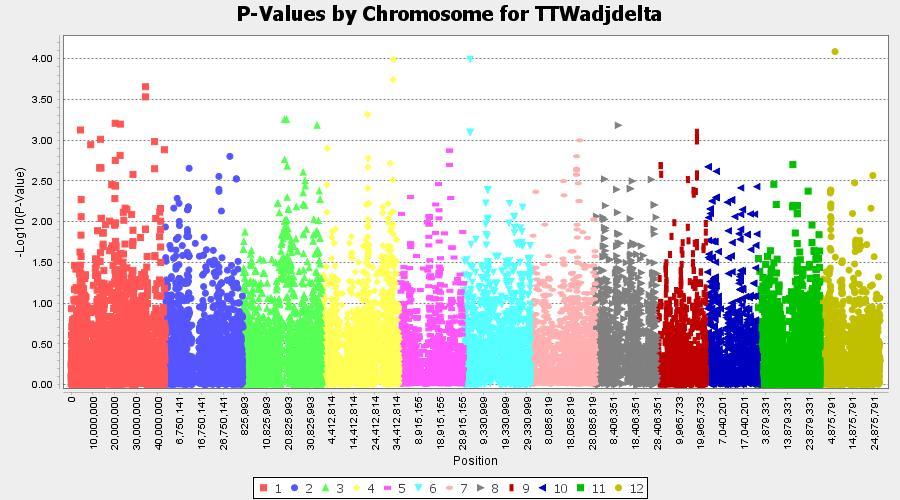  –log (P-value)  Chr1 2 3 4 5 6 7 8 9 10 11 12 | | Add 4. Fig 6.1.B 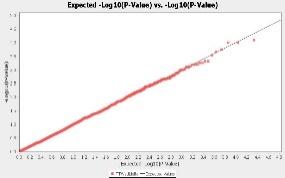  Expected –log (P-value) |
| Add 2. Fig 6.2.A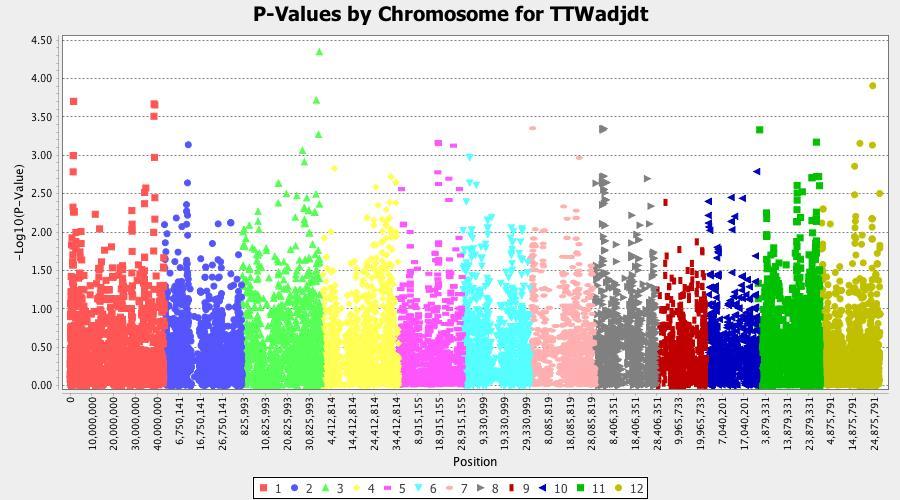  Expected –log (P-value)  –log (P-value)  Chr1 2 3 4 5 6 7 8 9 10 11 12 | | Add 2. Fig 6.2.B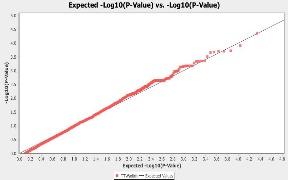 |
| Add 4. Fig 6.3.A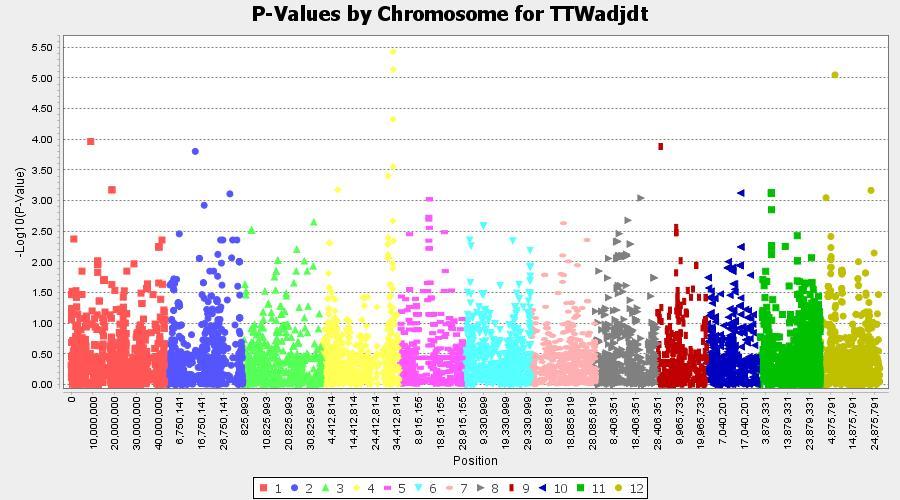  –log (P-value)  Chr1 2 3 4 5 6 7 8 9 10 11 12  **Additional file 4. Figure S6. GWAS on TTW** | | Add. Fig 6.3.B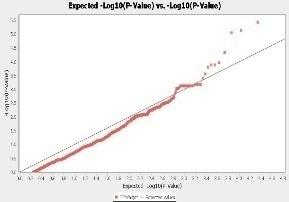  Expected –log (P-value) |
