# Supplementary figures and images for: Unraveling the Genetic Elements Involved in Shoot and Root Growth Regulation by Jasmonate in Rice Using a Genome-Wide Association Study
Source: Rice (N Y). 2019 Sep 4;12:69. doi: 10.1186/s12284-019-0327-5 (PMC6726733; doi:10.1186/s12284-019-0327-5)

## Slide 1
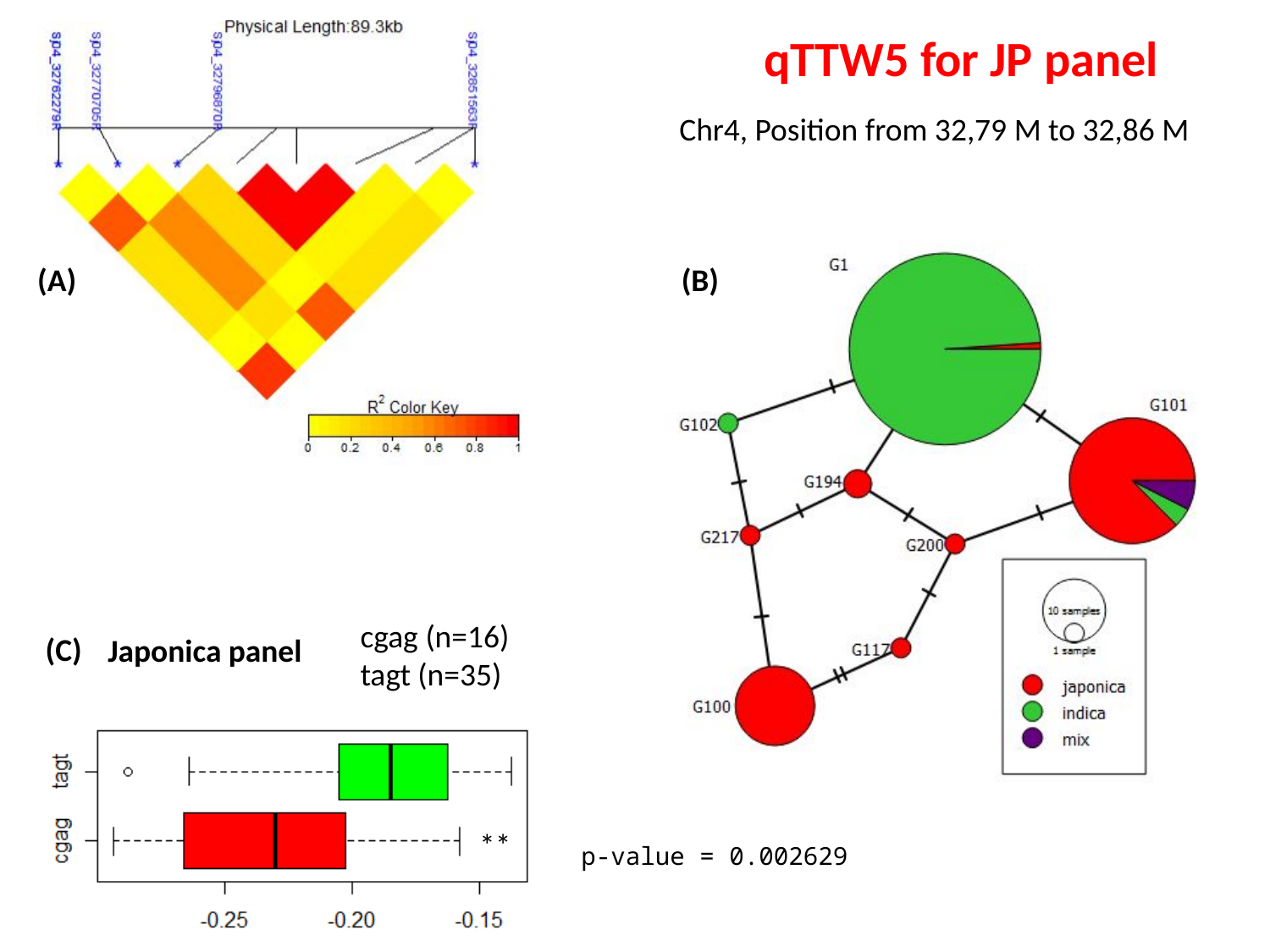

qTTW5 for JP panel
Chr4, Position from 32,79 M to 32,86 M
(A)
(B)
cgag (n=16)
tagt (n=35)
(C)
Japonica panel
**
p-value = 0.002629

Supplement: Supplementary file 6 — Figure S7. Haplotype analysis for qTTW5. (A) Linkage Disequilibrium heatmap in the peak region of association analysis GWAS for qRTW3. Significant SNP indicated as blue star in the photo and the pattern pairwise r2 of the associated SNPs in the QTL indicated with color code. Red color means SNPs are strongly associated to each other and yellow color means no association. The Linkage Disequilibrium heat map was created using the “LDheatmap” package in R (B) Population architecture of accessions based on the allelic combination significant SNPs in each QTL. Population architechture image is created by PopArt1.7 software . (C) Effect of allelic combination of 2 main haplotypes of each QTL on the value of interested traits. Number of accessions for each haplotype is indicated as (n). Welch Two Sample t-test was used to assess the differences between two haplotypes.*, **, *** indicated significant difference at p value < 0.05, 0.01 and 0.001 respectively. (PPTX 116 kb) [file 12284_2019_327_MOESM6_ESM.pptx]
